# Global identification of long non-coding RNAs involved in the induction of spinach flowering

**DOI:** 10.1186/s12864-021-07989-1

**Published:** 2021-09-30

**Authors:** Fatemeh Ghorbani, Reza Abolghasemi, Maryam Haghighi, Nematollah Etemadi, Shui Wang, Marzieh Karimi, Aboozar Soorni

**Affiliations:** 1 Department of Biotechnology, College of Agriculture, Isfahan University of Technology, Isfahan, Iran; 2 Department of Horticulture, College of Agriculture, Isfahan University of Technology, Isfahan, Iran; 3 College of Life Sciences, Shanghai Normal University, Shanghai, China; 4 Department of Plant Breeding and Biotechnology, College of Agriculture, University of Shahrekord, Shahrekord, Iran

## Abstract

**Background:**

Spinach is a beneficial annual vegetable species and sensitive to the bolting or early flowering, which causes a large reduction in quality and productivity. Indeed, bolting is an event induced by the coordinated effects of various environmental factors and endogenous genetic components. Although some key flowering responsive genes have been identified in spinach, non-coding RNA molecules like long non-coding RNAs (lncRNAs) were not investigated yet. Herein, we used bioinformatic approaches to analyze the transcriptome datasets from two different accessions Viroflay and Kashan at two vegetative and reproductive stages to reveal novel lncRNAs and the construction of the lncRNA-mRNA co-expression network. Additionally, correlations among gene expression modules and phenotypic traits were investigated; day to flowering was chosen as our interesting trait.

**Results:**

In the present study, we identified a total of 1141 lncRNAs, of which 111 were differentially expressed between vegetative and reproductive stages. The GO and KEGG analyses carried out on the cis target gene of lncRNAs showed that the lncRNAs play an important role in the regulation of flowering spinach. Network analysis pinpointed several well-known flowering-related genes such as *ELF*, *COL1*, *FLT*, and *FPF1* and also some putative TFs like MYB, WRKY, GATA, and MADS-box that are important regulators of flowering in spinach and could be potential targets for lncRNAs.

**Conclusions:**

This study is the first report on identifying bolting and flowering-related lncRNAs based on transcriptome sequencing in spinach, which provides a useful resource for future functional genomics studies, genes expression researches, evaluating genes regulatory networks and molecular breeding programs in the regulation of the genetic mechanisms related to bolting in spinach.

**Supplementary Information:**

The online version contains supplementary material available at 10.1186/s12864-021-07989-1.

## Introduction

Spinach (*Spinacia oleracea L.*) from the Amaranthaceae family is a beneficial annual vegetable species that is widely cultivated in different parts of the world and used in the human diet. It is a rich source of vital nutrients, containing *β*-carotene (provitamin A), vitamins of the B group, ascorbic acid, folates, and vitamin C. [1, 2]. Spinach is also known for its high iron content (4–6 mg per 100 g dry wt), and according to this feature, it is highly recommended for anemic people [3]. The dramatic increase in market demand has developed spinach breeding programs to introduce cultivars with a broader range of valuable traits, such as leaf texture, color, shape, pose, and petiole length. However, the development of cultivars to increase spinach production is positively related to other morphological traits such as plant height and bolting, induced by long-day exposure. Hence, the spring and summer spinach varieties have a greater tendency to bolting, causing a reduction in production [4–6]. Indeed, bolting is one of the most important productivity-related traits regulated by multiple signaling pathways and regulatory mechanisms [7, 8]. In recent years, studies on the identification of pathways and molecular functions of bolting-related genes have progressed in model plants, but few studies have focused on spinach [9–12]. However, the comparative transcriptome analysis of two spinach accessions with different bolting times identified genome-wide gene expression profiling and large-scale discovery of flowering-related genes from vegetative and reproductive leaves [13]. On the other hand, previous researches have revealed there are some non-coding RNA molecules (ncRNAs) that affect bolting and flowering by the regulation of genes expression [14, 15]. These ncRNAs are very heterogeneous in terms of their length, conformation and cellular function. In this regards, they can be separated into small non-coding RNA (small ncRNA) and lncRNA. Small ncRNAs are further divided into myriad subclasses, and each subclass has its own biological and medical importance. LncRNA can be further grouped into linear RNAs and circular RNAs (circRNA) types [16–20].

ncRNAs with more than 200 nucleotides are considered as lncRNAs, which originate from intronic and exonic regions of protein-coding genes in both sense and antisense strands, as well as from the intergenic regions [21]. Previous studies have demonstrated that lncRNAs act as one of the molecular mechanisms for the post-transcriptional regulation and modulation of protein function. Interestingly, lncRNAs have been shown to act as competing endogenous RNAs (ceRNAs), where miRNAs and lncRNAs regulate each other through their biding sites. Indeed, lncRNAs can promote gene expression by competing with miRNAs for specific binding sites in the non-coding regions of mRNAs and preventing the transcriptional repression caused by miRNAs [22, 23]. Hence, interactions of miRNA-mRNA, lncRNA-mRNA, miRNA-lncRNA, and lncRNA-mRNA-miRNA have been investigated by using various experimentally supported evidence or computationally predicted methods such as PceRBase [24] and LncACTdb [25].

LncRNAs are extremely found in diverse organisms and play critical functional roles in various biological processes, such as flowering time, root organogenesis, photomorphogenesis, and sexual reproduction [26–28]. Functional analysis of some lncRNAs has also indicated that they have potential roles in regulating temperature-dependent developmental changes, such as the transition from the vegetative to the reproductive phase and the bolting process. For example, FLINC is a lncRNAs, which plays a role in temperature-mediated flowering and is down-regulated at higher ambient temperature in *Arabidopsis* [29]. COLDAIR is another intronic lncRNA associated with the silencing and epigenetic repression of *FLOWERING LOCUS C* (*FLC*) to regulate flowering time in *Arabidopsis* [26]. Another lncRNA regulating developmental pathways known as long-day-specific male-fertility-associated lincRNA (LDMAR), which its expression lower than a certain level affects pollen development in rice under long-day conditions [30, 31]. Although a remarkable number of flowering-related lncRNA molecules have been identified by advanced sequencing technology in *Arabidopsis*, a large number of tissue-specifically expressed lncRNAs have also discovered in other plants such as strawberry [32], tomato [33], *Brassica rapa* [34], and *Coffea canephora* [35]. Based on RNA-seq datasets from different flower and fruit tissues of diploid strawberry, a large number of lncRNAs from various loci were identified and annotated [32]. According to the expression profile, 186 known lncRNAs, and 89 novel lncRNAs were found associated with pistil development in *Prunus mume*, which could provide new indications to elucidate how lncRNAs and their targets play role in pistil differentiation and flower development [36], highlighting the potential contributions of lncRNA in the flowering process. Although, for a better understanding of the functions lncRNAs and their target genes, several research works are carried out to identify abiotic stress responsive lncRNAs (In direct connection with flowering) under different conditions in many plants including *Camellia sinensis* [37, 38], *Capsicum annuum* [39], *Mangifera indica* [40], *Arachis hypogaea* Linn [41], *Zea mays* [42], a substantial number of databases have been also developed to provide resources and broadly investigate lncRNAs in plants [20, 43–45].

Therefore, there is a critically important role for lncRNAs to control flowering time in various crops, especially for the sensitive plants to bolting. Hence, we characterized lncRNAs in two different spinach accessions (Kashan and Viroflay) at the transcriptome level and compared their expression levels before and after flowering. According to the results of previous research, the vegetative characteristics of 44 spinach accessions [46], two accessions Viroflay and Kashan were placed in the group of late and early flowering spinach, respectively. Indeed, maximum variation for the trait of “days to flowering” was found between accessions Viroflay (87 days) and Kashan (43 days). Thus, we performed transcriptome and qPCR analyses to reveal lncRNAs and hub genes associated with spinach bolting in two accessions with different bolting time. To further examine the role of lncRNAs in bolting, we also constructed a co-expression network using weighted gene co-expression network analysis (WGCNA) based on differentially expressed mRNAs and lncRNAs. Moreover, the relationship between modules and their correlation with the stages of each accession was detected. In our experiment, a robust and complete set of lncRNAs were identified, and the basic features of these lncRNAs were also characterized. We also obtained differentially expressed lncRNAs (DELs) from different stages of two accessions by differential expression analysis. Finally, by conducting gene co-expression network analysis, we identified several functional modules correlated to bolting. Overall, our results provide the basis for future studies on lncRNAs activity mechanisms in spinach bolting.

## Results

### Novel lncRNAs identification

**Fig. 1:**
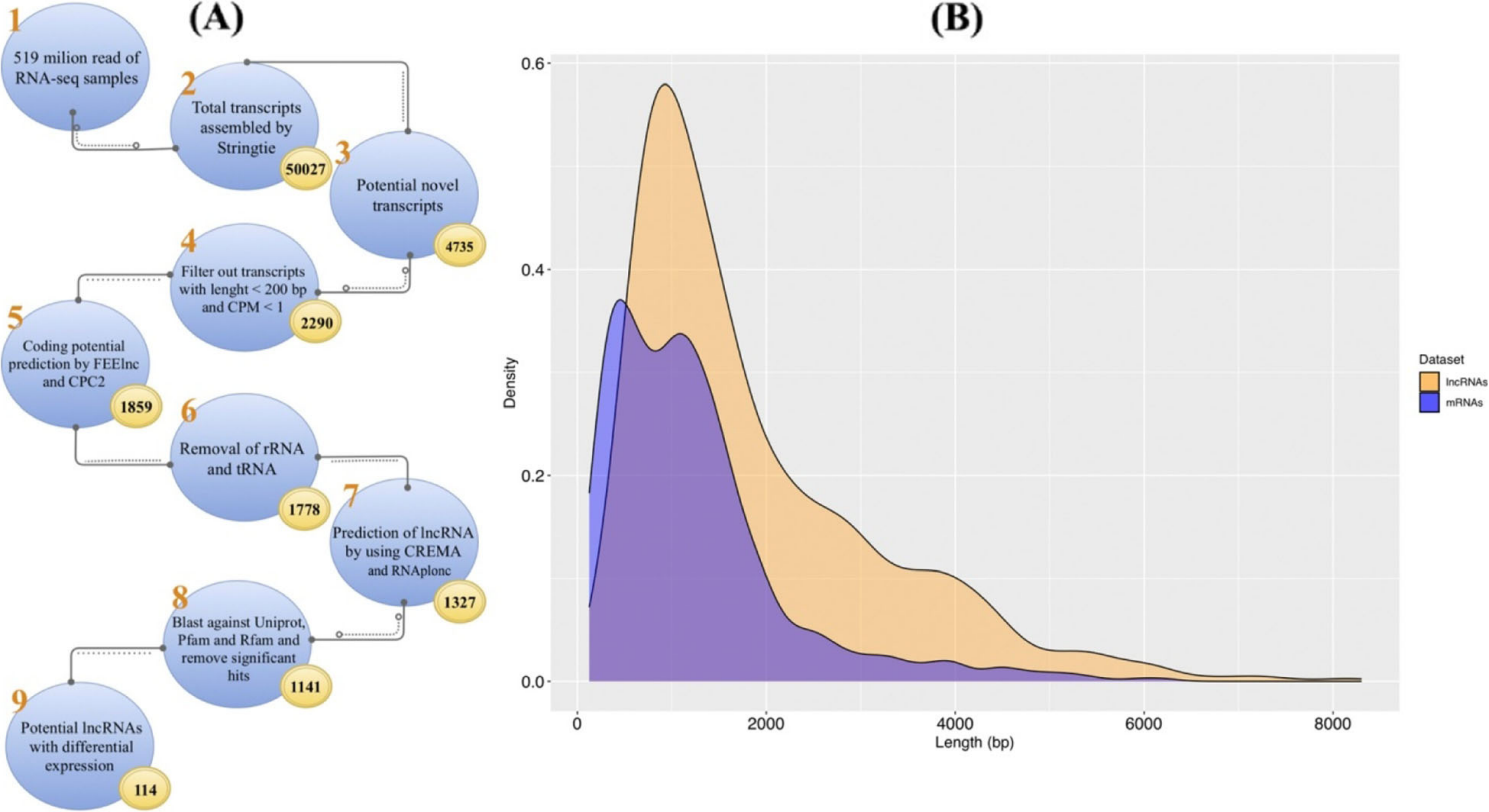
Identification and characterization of lncRNAs in spinach. (**A**) Detailed flow diagram of the bioinformatics pipeline for the identification of lncRNAs. Different filters were applied for the identification of lncRNAs; numbers representing the total number of transcripts identified at each filter. (**B**) Length distribution of spinach lncRNAs

**Fig. 2:**
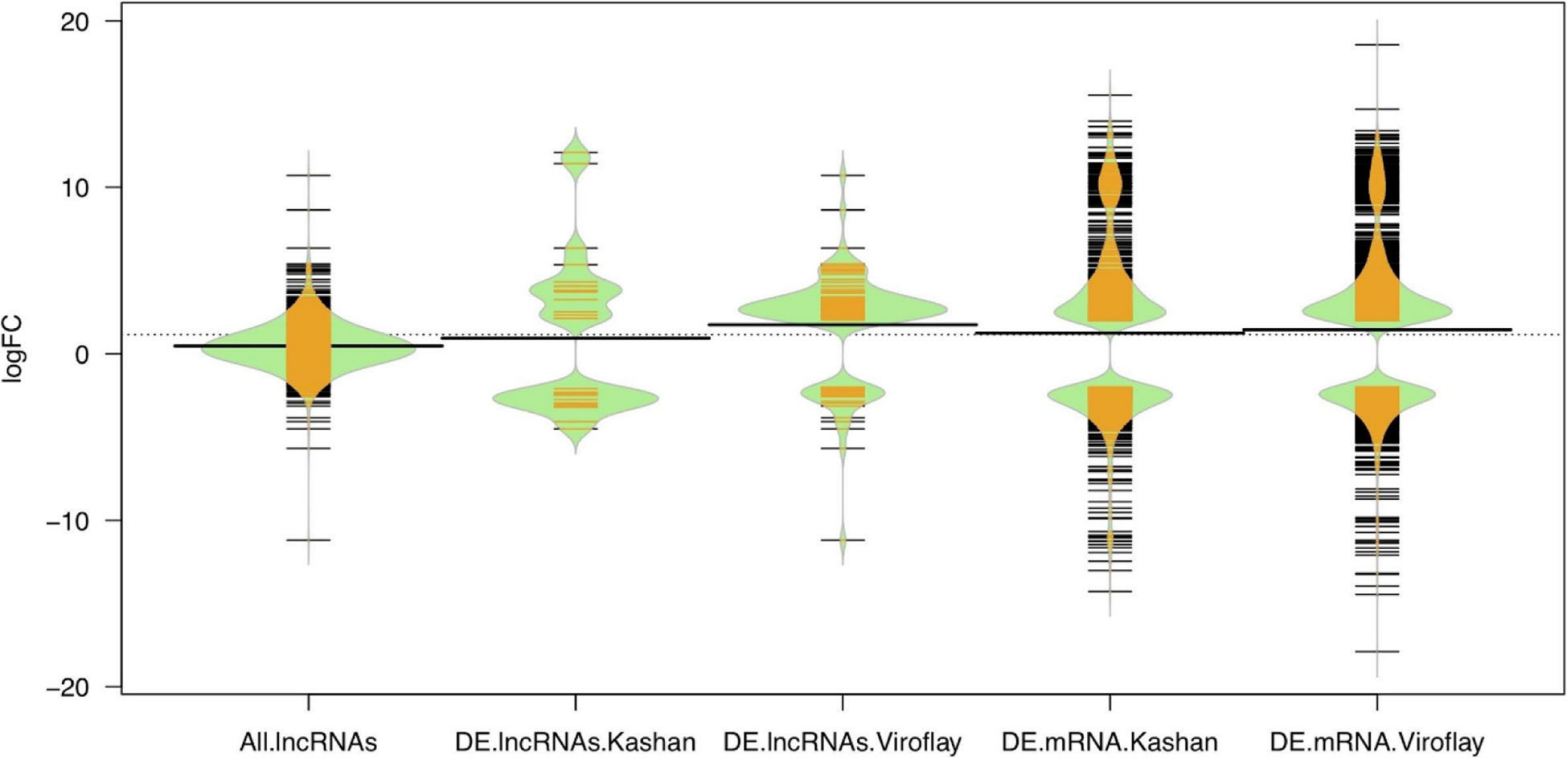
Bean plots of differential expression levels (log-fold change) of lncRNAs and coding genes (mRNAs) detected in each accession under stages (vegetative and reproductive) comparison. DE represents differentially expressed genes

According to the lncRNAs identification pipeline (Fig. 1 A), after reconstructing the transcriptome for each RNA-Seq library and combining assemblies, a total of 50,027 transcripts were identified through Stringtie software for both accessions. Of those, 4735 transcripts were identified as unannotated transcripts. The GFF, BED (identifier and genomic locations), and FASTA files of all the unannotated transcripts are provided in DataS1. From the total unannotated transcripts, 1925, 324, 37, and 4 transcripts with class codes ‘u’ (intergenic), “o” (generic overlap with known exon), “i” (intronic), and “x” (overlap with a known gene on the opposite strand) were expressed with CMP > 1. Afterward, FEELnc identified 2230 lncRNAs among remaining unannotated transcripts. Then, we assessed the protein-coding potential of transcripts using the CPC program and deleted 371 potential coding transcripts. The steps to filter tRNA and rRNAs removed 81 transcripts. Transcripts were then inputted into CREMA[47] (https://github.com/gbgolding/crema) for lncRNAs prediction. Utilizing CREMA’s numerical scoring system for lncRNAs prediction, 1327 transcripts with a prediction score > 0.5 were considered putative lncRNAs. Using a rigorous filtering pipeline to remove transcripts with any known protein domains documented in the Pfam database, housekeeping RNAs such as tRNAs, rRNAs, snRNAs, and snoRNAs in the Rfam database, and encoding any conserved protein, 186 transcripts were detected and filtered out from the further analysis. In total, 1141 transcripts were identified as lncRNAs in the spinach transcriptome. A file containing all the identified lncRNAs sequences, along with their genomic locations, is provided in DataS2. We further counted the length distribution (Fig. 1B) and characterized the subgenome and chromosome location of lncRNAs. The results indicated that the length distribution of the lncRNAs ranged from 222 bp to 8,296 bp. The majority of lncRNAs ( approximately 65.6 %) were found in a range of 222 to 2,000 nucleotides, while 30.8 % of lncRNAs had a size between 2000 and 5,000 bp, and only 3.5 % had length over 5,000 bp. In addition, we found that the lengths of lncRNAs were largely varied than their target genes. Generally, target mRNAs lengths varied from 126 to 6,207 bp and also distributed in all spinach chromosomes (Fig. 1B). It was also found that 43.6 % lncRNAs were distributed across six spinach chromosomes, and the highest densities of lncRNAs were detected in chromosome numbers 3 and 4. The comparison result between spinach lncRNAs and lncRNAs available in the above-mentioned databases indicated that more spinach lncRNAs appear to be extensively conserved. Total 17 putative spinach lncRNAs had at least one significant hit in CANTATAdb and PLncDB databases [43, 45] from three plant species (Table S1) including *Chenopodium quinoa*, *Solanum lycopersicum*, and *Trifolium pratense*. Developmental stages comparison in each accession revealed 28 and 97 lncRNAs as significant differentially expressed lncRNAs (DE-lncRNAs) in accessions Kashan and Viroflay, respectively. Among them, 11 transcripts were shared between accessions (Fig. S1; DataS3). Among unique DE-lncRNAs, 8 and 67 genes were up-regulated in the reproductive stage of Kashan and Viroflay. By comparison of log fold change (logFC) of DE-mRNAs and DE-lncRNAs in both accessions, we found that lncRNAs were expressed at different levels in the Kashan and Viroflay (Fig. 2). Interestingly, the overall expression changes in mRNAs were higher than lncRNAs.

### Functional analysis of the lncRNAs

**Fig. 3:**
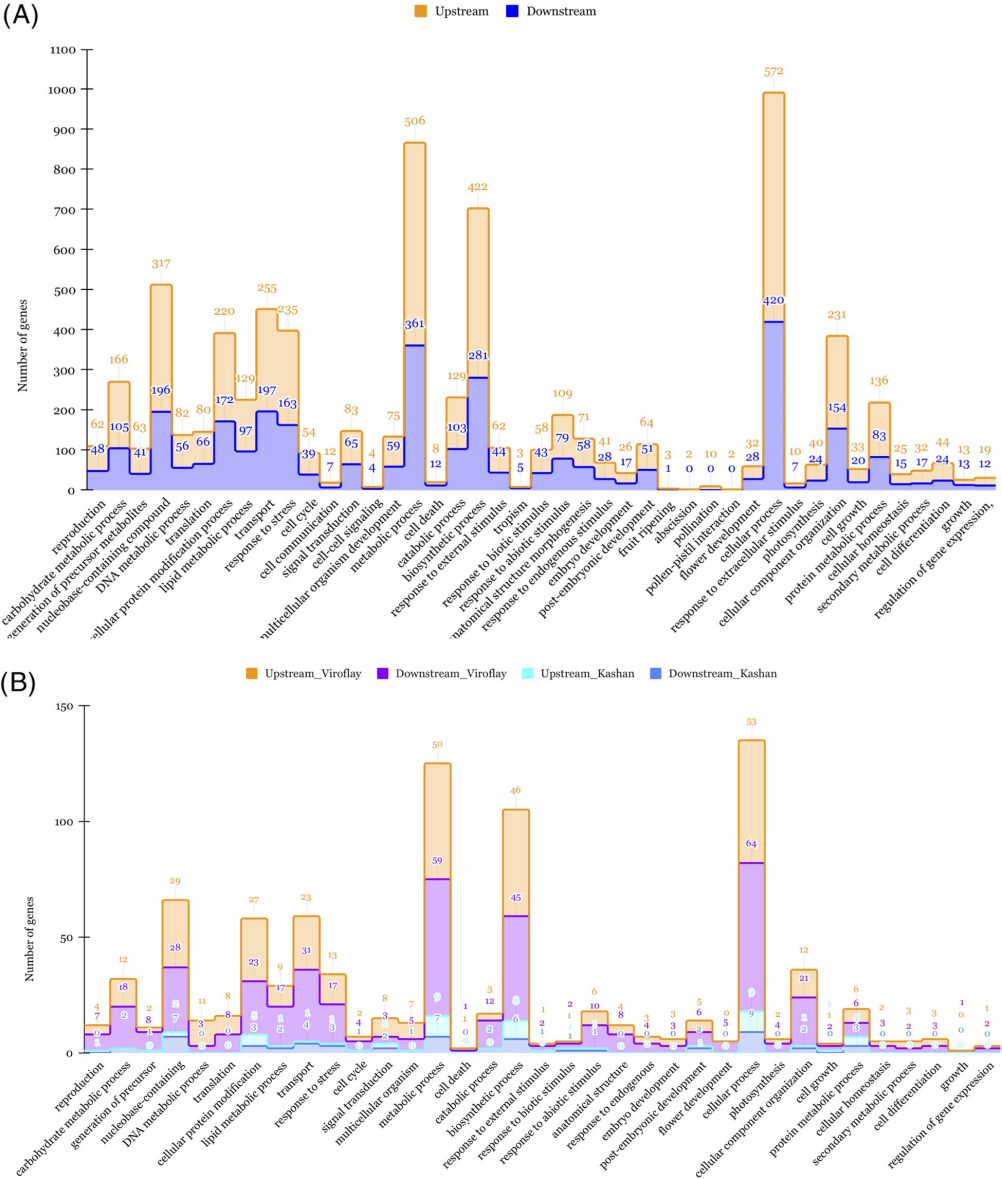
Gene classification for protein-coding genes spaced less than 100 kb away from the upstream and downstream of all lncRNAs (**A**) and DE-lncRNAs (**B**) detected in each accession under stages (vegetative and reproductive) comparison. The histogram shows the classification of genes under the biological process category. Values on each bar represent the number of genes identified in that category

**Fig. 4:**
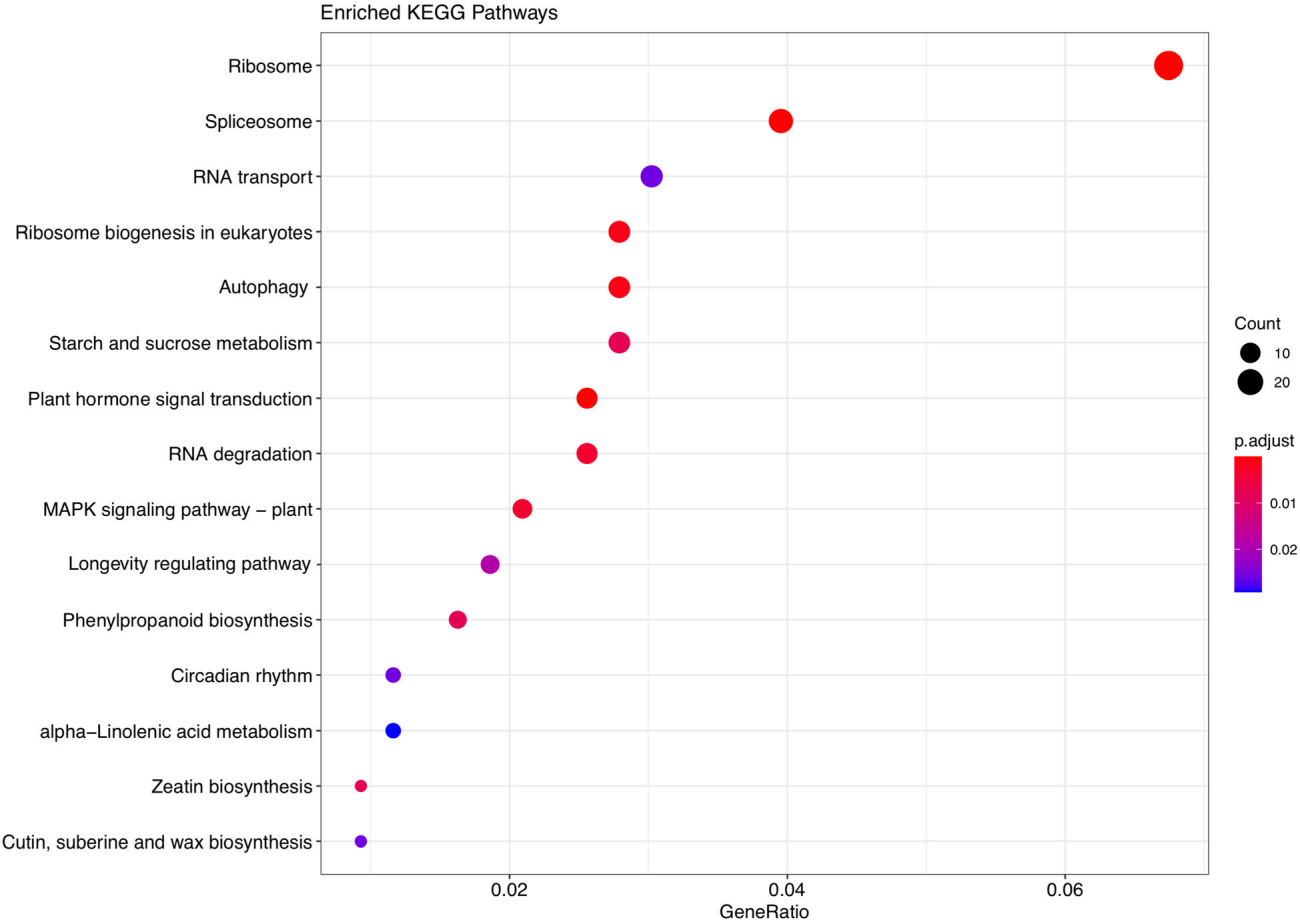
KEGG pathway enrichment analysis of target genes within 100 kb upstream and downstream sites of all identified lncRNAs. The x-axis represents the ratio number of target genes, and the y-axis displays the KEGG pathway terms. The size of the bubble shows the enrichment significance, while colors indicate the enrichment score

Since lncRNAs located upstream and downstream of protein-coding genes may be involved in regulatory activities (cis and trans roles), we searched protein-coding genes from 100 kb upstream and downstream of potential lncRNAs to explore their function in the cis role. By screening 100 kb upstream and downstream sites of all identified lncRNAs, we detected 2715 and 1973 target genes, respectively. As a result, we found that among upstream target genes, 66 and 149 genes were DEGs between stages in accessions Kashan and Viroflay, respectively. According to the results of downstream target genes, 32 and 99 DEGs were screened in accessions Kashan and Viroflay, respectively. We further found that DE-lncRNAs in Kashan and Viroflay were neighbored to 42 and 270 coding genes. Furthermore, there were two DEGs among the target genes of DE-lncRNAs in Kashan, while 40 DEGs were found among the target genes of DE-lncRNAs in Viroflay. Subsequently, to predict and classify possible functions of lncRNAs, all target genes were aligned to GO terms using Gene Classification tools located in SpinachBase [48]. According to the details of the GO analysis, of the 2715 target genes located upstream of lncRNAs, 1423, 1402, and 1280 were successfully annotated with GO assignments in the three main categories, including biological process (BP), molecular function (MF), and cellular component (CC), respectively. The counterpart numbers of target genes derived from the downstream of lncRNAs were 1053, 995, and 765. Concerning the 42 target genes located upstream and downstream of DE-lncRNAs in accession Kashan, BP was the dominant category with 23 genes, followed by MF with 18 and CC with 12 genes. Concerning the accession Viroflay, 138, 139, 87 genes were involved in different GO terms of BP, MF, and CC categories, respectively. To obtain a deeper understanding, the further analysis covered only the GO terms associated with biological processes (Fig. 3). Enrichment results exhibited that functional subcategories, including ‘cellular processes’, ‘metabolic processes’, and ‘biosynthetic processes’ were dominant under the biological processes. Besides that, a significant number of target genes were classified into reproduction (GO:0000003), carbohydrate metabolic process (GO:0005975), and flower development (GO:0009908) subclasses, which are related to the inflorescence development activities, flowering and bolting regulation, and signaling molecules. Additionally, we found GO terms related to embryo development (GO:0009790), post-embryonic development (GO: 0009791), and pollination (GO: 0009856), which are well known to be involved in the transition from the vegetative stage to the flowering stage. Compared to specific biological processes for accession Viroflay and Kashan, the most GO terms were dominated in Viroflay. Besides GO analysis, the KEGG enrichment analysis results showed that the target genes of lncRNA were mainly enriched in several flowering-related pathways, including “Starch and sucrose metabolism”, “MAPK signaling pathway”, “circadian rhythm”, and “phenylpropanoid biosynthesis” (Fig. 4).

### Assessment of conservation and expression of identified lncRNAs in the spinach flower tissues

**Fig. 5:**
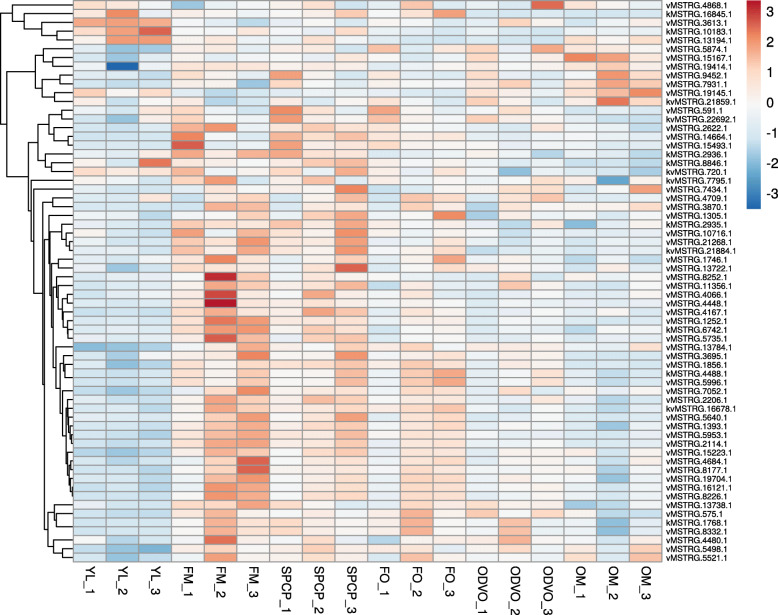
The heatmap of DE-lncRNAs identified in flower tissues. Rows: flower samples; columns: LncRNAs; colour key indicates LncRNA expression value,

In further assessment, we examined the conservation and expression of identified lncRNAs in flower tissues through identification and expression analysis of a flower transcriptome dataset with five different developmental stages including, FO, SPCP, FM, ODVO, and OM. According to the results, from 1141 lncRNA identified in this study, 768 (~ 68 %) were found in flower tissue, of which 8 and 50 were DE-lncRNA in Kashan and Viroflay respectively. Furthermore, 6 common DE-lncRNAs between both accessions were found to be expressed in flower tissue. The expression profile of these lncRNAs across all the flower-related samples along with the young leaf-related ones was depicted in the heatmap plot (Fig. 5). As the plot is shown, lncRNAs exhibited a varied expression pattern during different developmental stages. The majority of 64 DE-lncRNAs displayed overexpression in the “FM” stage which among them vMSTRG.8252.1, vMSTRG4066.1, and vMSTRG4448.1 indicated the highest level of expression.

On the basis of differential expression analysis, kMSTRG.16845.1 was found as a dominant DE-lncRNA between different stages (Table 1). The significant down expression of this lncRNA was observed in the late stages (“ODVO” and “OM”) of flower development compared to the early stages (“FO” and “SPCP”) and also “YL” stage. Similarly, the significant down-regulation of kMSTRG.8846.1 was detected in “OM” compared to “YL”, “SPCP” and “FM” stages. Indeed, our results suggested the potential negative regulatory role of kMSTRG.16845.1 and kMSTRG.8846.1 in flower development processes. The expression of kMSTRG.10183.1 showed a down-regulation pattern in “FO” and “ODVO” compared to “YL”. Another flower-related lncRNA, vMSTRG.15493.1, appeared with a positive regulatory role in the development of spinach flower because of its significant overexpression in each of “FM”, “FO”, and “SPCP” compared to “YL” stage. Likewise, vMSTRG.13194.1 was significantly up-regulated in each of “ODVO” and “SPCP” compared to “YL”. Additionally, its significant overexpression in the latest stage (“OM”) in comparison with “FO” and “ODVO” was observed.

**Table.1:** DE-lncRNAs identified in leaf tissue which differentially expressed in flower tissues

IDs	logFC	Regulation
kMSTRG.16845.1_FMvsODVO	-2.261436098	UP_FM_DOWN_ODVO
kMSTRG.16845.1_FMvsOM	-2.598384798	UP_FM_DOWN_OM
kMSTRG.8846.1_FMvsOM	-2.055652066	UP_FM_DOWN_OM
kMSTRG.16845.1_FOvsODVO	-3.055670498	UP_FO_DOWN_ODVO
kMSTRG.16845.1_FOvsOM	-3.410995383	UP_FO_DOWN_OM
kMSTRG.16845.1_SPCPvsODVO	-2.774997488	UP_SPCP_DOWN_ODVO
kMSTRG.8846.1_SPCPvsOM	-2.575980048	UP_SPCP_DOWN_OM
kMSTRG.16845.1_SPCPvsOM	-3.115322939	UP_SPCP_DOWN_OM
kMSTRG.10183.1_YLvsFO	-2.073944301	UP_YL_DOWN_FO
kMSTRG.10183.1_YLvsODVO	-2.152235277	UP_YL_DOWN_ODVO
kMSTRG.16845.1_YLvsODVO	-3.776583683	UP_YL_DOWN_ODVO
kMSTRG.16845.1_YLvsOM	-4.095162324	UP_YL_DOWN_OM
kMSTRG.8846.1_YLvsOM	-3.030554633	UP_YL_DOWN_OM
vMSTRG.13194.1_FOvsOM	2.181675592	DOWN_FOR_UP_OMR
vMSTRG.13194.1_ODVOvsOM	2.229283368	DOWN_ODVOR_UP_OMR
vMSTRG.15493.1_YLvsFM	2.182436621	DOWN_YLR_UP_FMR
vMSTRG.13194.1_YLvsFO	-3.271367298	UP_YLR_DOWN_FOR
vMSTRG.15493.1_YLvsFO	2.05220628	DOWN_YLR_UP_FOR
vMSTRG.13194.1_YLvsODVO	-3.315337476	UP_YLR_DOWN_ODVOR
vMSTRG.15493.1_YLvsSPCP	2.453248357	DOWN_YLR_UP_SPCPR

### Assessment of DE-lncRNA-DEG interaction using LncTar

LncRNAs can directly interact with their complementary mRNA transcripts by base-pairing [49]. To investigate whether the neighbouring loci are trans-regulated by lncRNAs, we employed lncTar (http://www.cuilab.cn/lnctar) on 28 DE-lncRNAs and 42 up/down stream adjacent DEGs detected in Kashan, as well as on 97 DE-lncRNAs and 270 upstream and 293 downstream neighbouring DEGs in Viroflay. According to the results, in order, 7 and 8 upstream and downstream DEGs were identified as potential targets of 10 and 11 DE-lncRNAs in Kashan respectively. Regarding the annotation of predicted lncRNA trans-regulated target genes, putative transcription factor PHYTOCHROME INTERACTING FACTOR 4 (*PIF4*:Spo08670) was found as a flowering-time-related gene among downstream adjacent DEGs in Kashan. This gene was predicted to be targets for 11 DE-lncRNAs, 6 of which were found to be conserved and expressed in flower tissues during flower developmental stages. In Viroflay, 30 and 32 DE-lncRANs were detected to target 54 and 56 upstream and downstream DEGs respectively. Regarding the annotation of trans target genes located in downstream of DE-lncRNAs in Viroflay, Serotonin *N*-acetyltransferase (SNAT2:Spo03879), Enhancer of AG-4 1 (*HUA1*:Spo21977), and *EARLY FLOWERING 6* (*ELF6*: Spo26619) were detected as flowering-related genes among downstream neighbouring DEGs. Interestingly, it was found DE-lncRNAs that potentially regulate these three target genes differentially expressed in flower tissue between different flower developmental stages.

According to the annotation of predicted target genes, 9 upstream DEGs were directly involved in flowering. These DEGs included CHD3-type chromatin-remodeling factor PICKLE (*PKL*:Spo01259), Protein LEAFY (*LFY*:Spo05659), E3 ubiquitin-protein ligase ORTHRUS 2 (*ORTH2*:Spo05660), Casein kinase II subunit beta-3 (*CKB3*:Spo06583), PHYTOCHROME-DEPENDENT LATE-FLOWERING (*PHL*:Spo09998), Gibberellin-regulated protein 5 (*GASA5*: Spo11995), Folylpolyglutamate synthase (*FPGS1*: Spo15152), MEDIATOR 13 (*MED13*:Spo15416) and WD-40 repeat-containing protein MSI4 (*MSI4*: Spo19562). All above flowering-related genes were predicted to be targets of 13 DE-lncRNAs, 7 of which were recognized as DE-lncRNAs in flower tissue among flower developmental stages as well. Interestingly, In Kashan and Viroflay accessions, all predicted flowering-related target genes were found to be regulated by 11 common DE-lncRNAs which can demonstrate the close association of these genes in pathways controlling induction of spinach flowering.

### LncRNA act as endogenous target mimics (eTMs) of miRNAs

In the present study, 66 DE-lncRNAs were identified as eTMs of 65 miRNAs (Table S2). Of these, 7 and 54 DE-lncRNAs were found in accessions Kashan and Viroflay, respectively, while 5 DE-lncRNAs were commonly shared between accessions. The results showed that a total of 61 miRNAs had various target mRNAs ranging from one to 437 genes. It was also noted that two DE-lncRNAs, named MSTRG.16566.1 (chr4:76,795,449–76,796,456) and MSTRG.16121.1 (chr4:38,264,207–38,266,517), were predicted to be potential eTMs for miR172, and miR167. Among the predicted targets of miR-172 (14 targets), we found three genes encoding AP2/ERF transcription factors (TFs).

### Co-expression Network Analysis

**Fig. 6:**
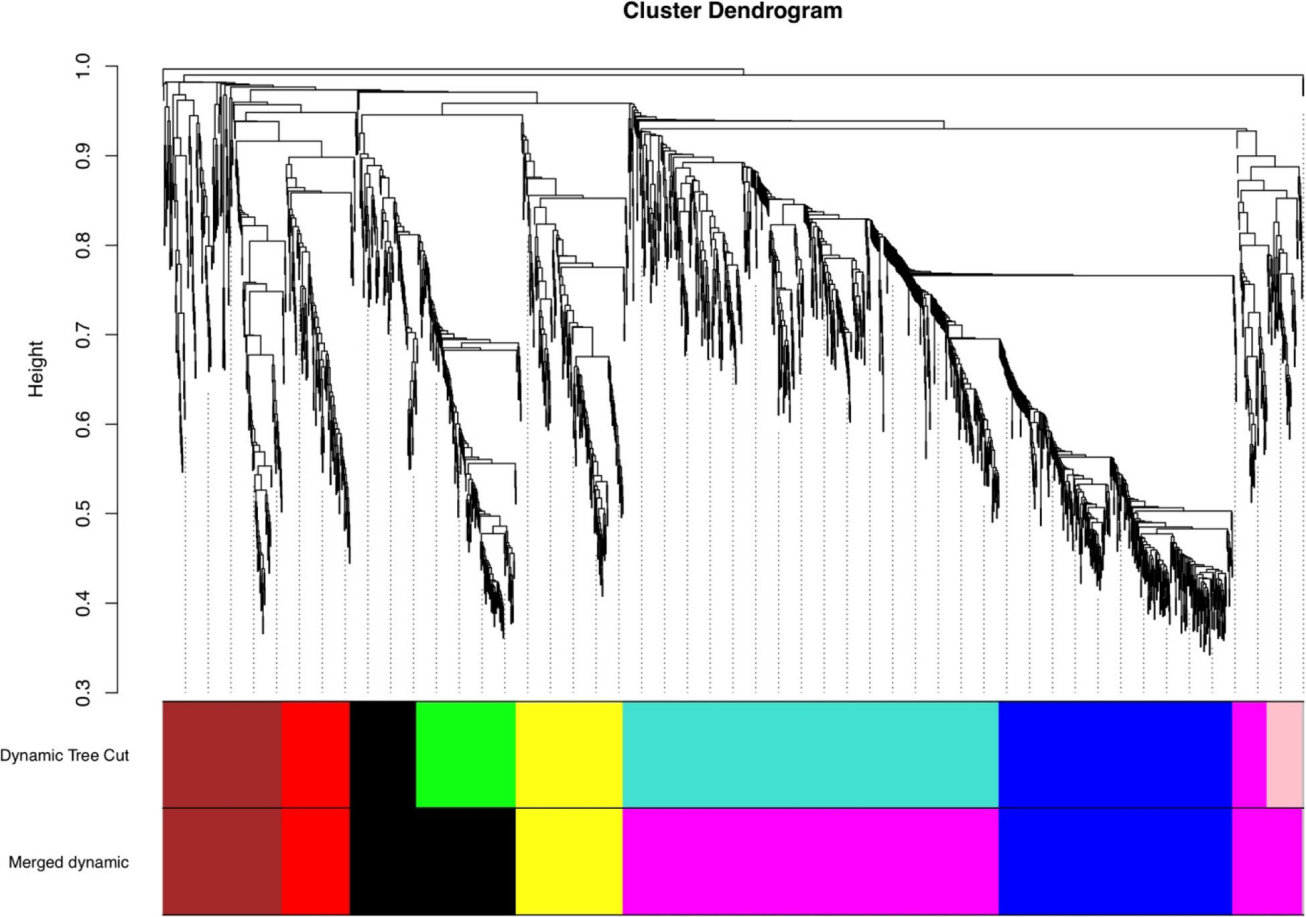
The modules identified by WGCNA. Gene hierarchical cluster dendrogram based on a dissimilarity measure of the Topological Overlap Matrix (1-TOM) calculated by WGCNA. The branches correspond to modules of highly interconnected groups of genes. Two colored bars below the dendrogram represent the original modules and merged modules

It is documented if lncRNAs indicate similar expression patterns with some mRNAs, those lncRNAs can be supposed as regulators of target mRNAs [28, 50, 51]. Hence, the read count data belonging to differentially expressed genes obtained from RNA-Seq of six Kashan and six Viroflay samples as early and late-bolting accessions were used to construct the co-expression network. In this approach, we identified 9 modules (clusters of highly co-expressed genes) (Fig. 6), labeled by black, blue, brown, green, red, turquoise, pink, magenta, and yellow, containing 61 to 668 genes in magenta and turquoise modules, respectively.

These modules were composed of dozens to hundreds of mRNAs, with a varied ratio from 1.6 % (pink) to 18.8 % (green) between DE-lncRNAs and mRNAs (Table 2). The turquoise module contained the largest number of DE-lncRNAs (32 DE-lncRNAs), followed by the green module containing 28 DE-lncRNAs.

**Table.2:** The number of genes per module and the percentage of DE-lncRNAs in each module

Module name	Number of mRNAs	Number of DE-lncRNAs	% of DE-lncRNAs
Turquoise	636	32	5.0
Blue	395	20	5.1
Brown	202	9	4.5
Yellow	178	12	6.7
Green	149	28	18.8
Red	115	6	5.2
Black	113	5	4.4
Pink	62	1	1.6
Magenta	60	1	1.7

**Fig. 7:**
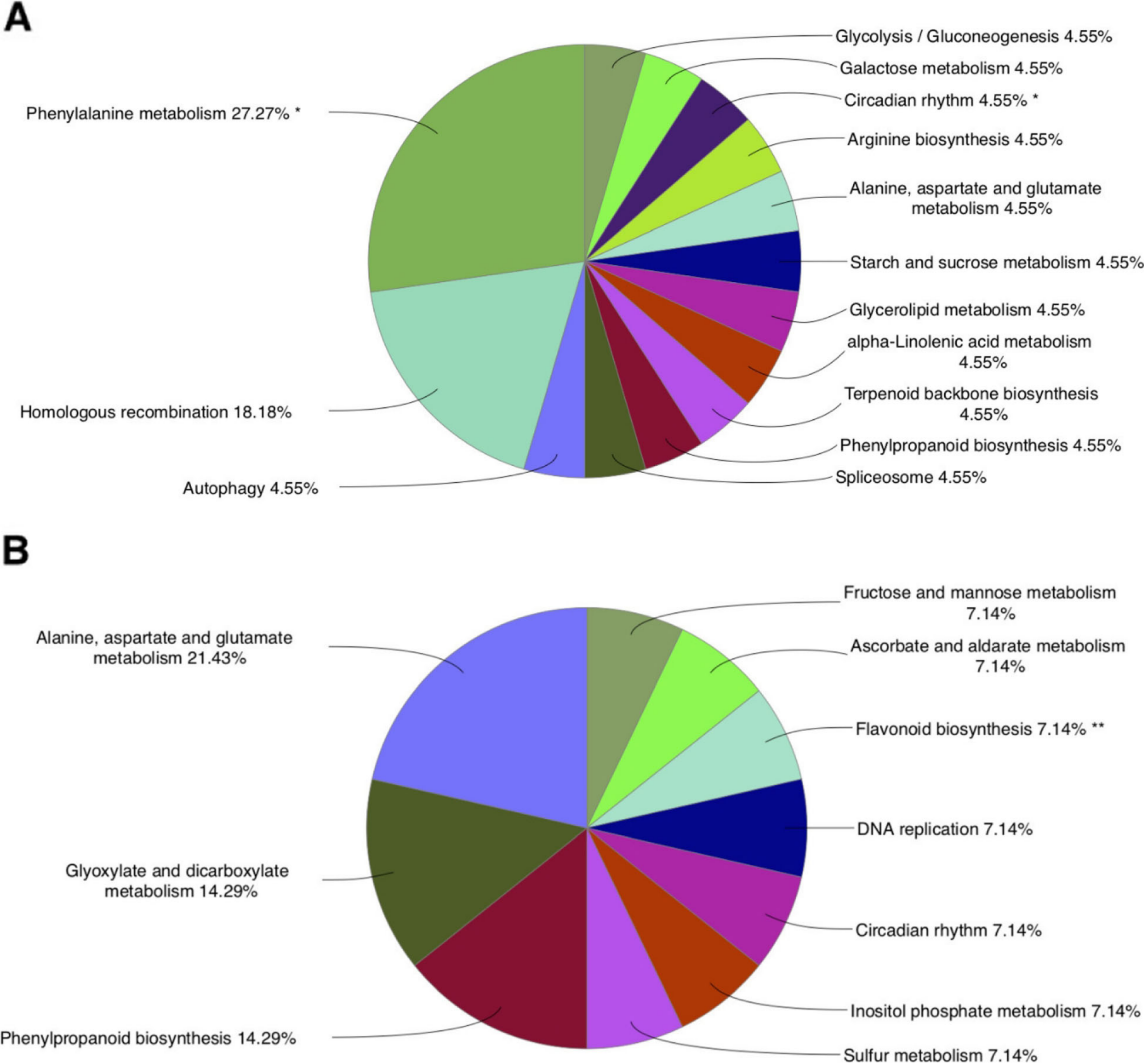
The top enriched KEGG pathways of genes in turquoise (**A**) and blue (**B**) modules

To reveal enriched pathways contributed by each co-expressed module, we performed KEGG analysis of DEGs from each module separately. Through KEGG functional enrichment analysis, we found only genes in the turquoise and blue modules were significantly enriched in 14 and 11 pathways (Fig. 7). Among the top pathways, phenylpropanoid biosynthesis and circadian rhythm were common enriched pathways between both modules. Regarding the role of carbohydrates in flowering and inflorescence development, we found several carbohydrate-related pathways, including “starch and sucrose metabolism” and “galactose metabolism” in the turquoise module and “fructose and mannose metabolism” in the blue module. These carbohydrate-related pathways were equally enriched with “circadian rhythm” in its correspondent module, suggesting that they are engaged in the flowering pathway. In previous researches, the exploration of flower differentiation-related genes and the information of carbohydrate metabolism that are related have been fully analyzed. Indeed, previous studies using the different samples at different stages of various plant species have indicated that proteins involved in carbohydrate metabolism are responsive to flowering time [52–55].

**Fig. 8:**
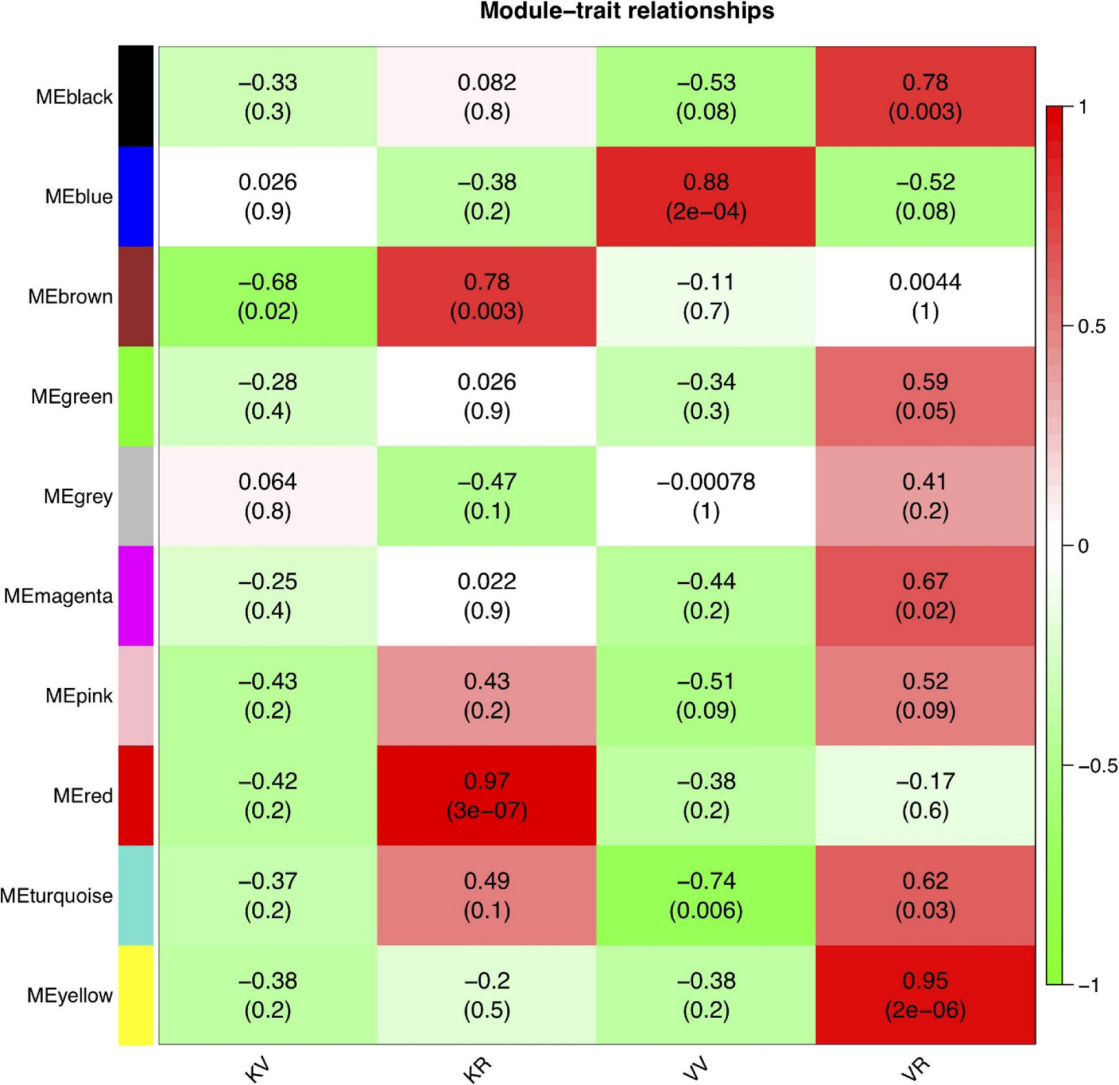
Matrix showing the module-trait relationship of different co-expression modules and external traits. Each row corresponds to a module eigengene, column to a trait. KV, KR, VV, and VR represent Kashan-vegetative, Kashan-reproductive, Viroflay-vegetative, and Viroflay-reproductive, respectively. The numbers represent the Pearson correlation coefficient values and *P-values*. Red and green color represent positive and negative correlations, respectively. The table is color-coded by correlation according to the color legend

To understand the relationship between modules and external traits, we performed Pearson correlation coefficient analysis to connect each of the co-expression modules with each stage and accession (Fig. 8). It was observed that most modules had a positive correlation with the reproductive stage of both accessions, suggesting that genes in these modules may positively regulate flowering in spinach. Thus, most of these genes should be up-regulated in the transition from the vegetative stage to the flowering stage. The red and brown modules (with r = 0.97 and r = 0.78) highly correlated with the reproductive stage of accession Kashan; in contrast, yellow and black modules (with r = 0.95 and r = 0.78) had a positive correlation with the reproductive stage of accession Viroflay. Among modules, only one co-expression module, the blue module, specifically correlated (*r* = 0.88) with the vegetative stage of accession Viroflay.

**Fig. 9:**
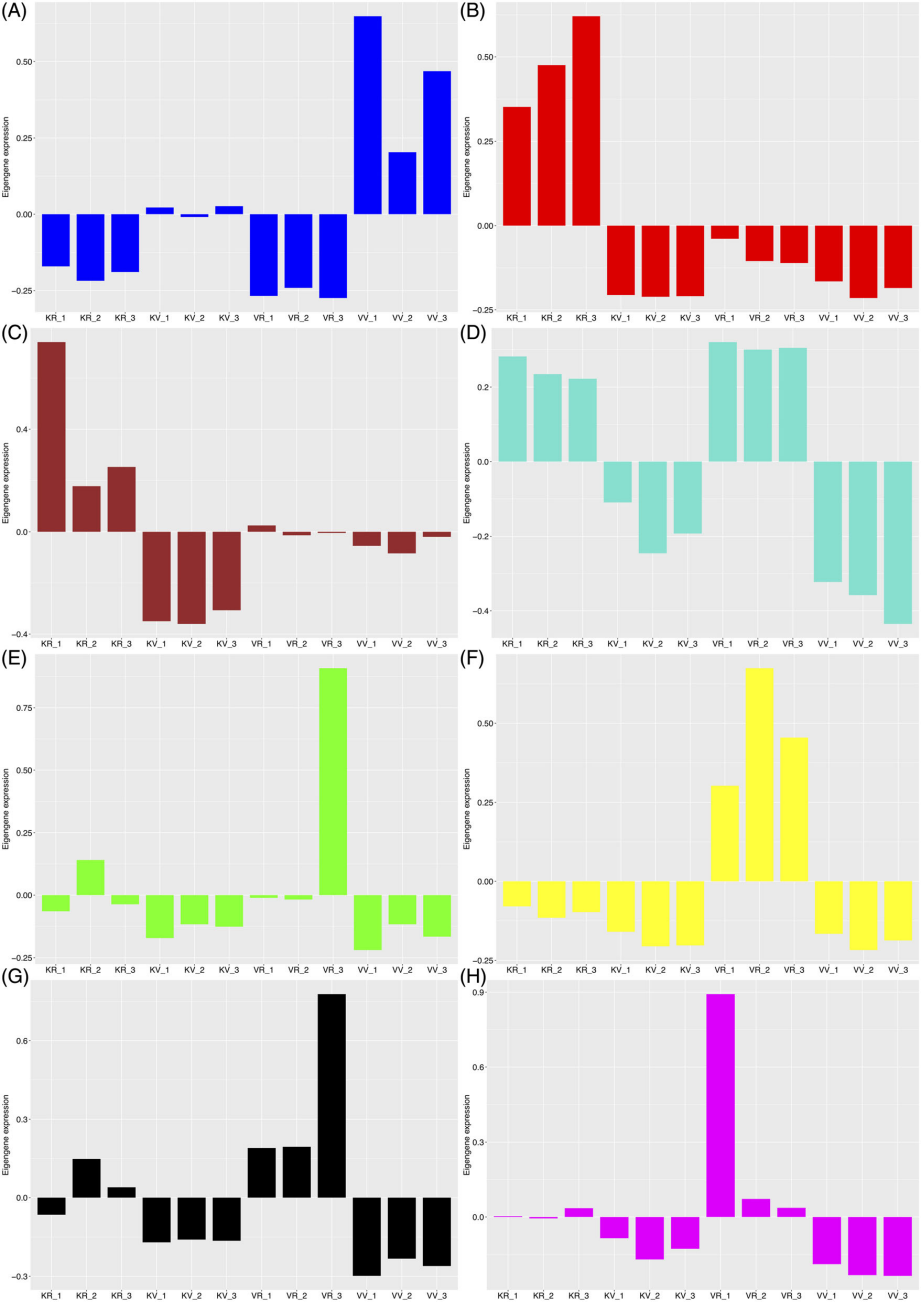
Profile of eight major modules, including (**A**) blue, (**B**) red, (**C**) brown, (**D**) turquoise, (**E**) green, (**F**) yellow, (**G**) black, and (**H**) magenta. The y axis indicates the value of the module eigengene, the x-axis the sample type. KV, KR, VV, and VR represent Kashan-vegetative, Kashan-reproductive, Viroflay-vegetative, and Viroflay-reproductive, respectively. The numbers beside each sample represent replicate

To further understand each co-expressed module’s particularity concerning their expression patterns in the different datasets, we plotted eigengene expression values of the genes belonging to each module along with each sample. According to these results, we observed that in all detected modules except the blue module and some replicates in the brown (KV_2 and KV_3), the gene expression levels were higher in the reproductive stage of both accessions than in the vegetative stage (Fig. 9). The blue and yellow modules (Fig. 9 A, F) genes appeared to be more relative in expression to the vegetative and reproductive stages of Viroflay as a late-bolting accession, confirming the positive correlations observed earlier in Pearson correlation coefficient analysis between modules and stages in each accession. In contrast, the red and brown modules (Fig. 9B, C) genes displayed a similar trend with more induction in the reproductive stage of Kashan as an early-bolting accession. According to obtained results, the turquoise module genes (Fig. 9D) appeared to be modulated in the vegetative stage of both accessions through down-regulation, whereas in the black module (Fig. 9G) genes were positively regulated in the reproductive stage of Viroflay, suggesting genes in this module play important central and key roles in regulating the flowering time of Viroflay as a late-bolting accession.

### Networks displaying relationships among genes and lncRNAs within co-expressed modules

**Fig. 10:**
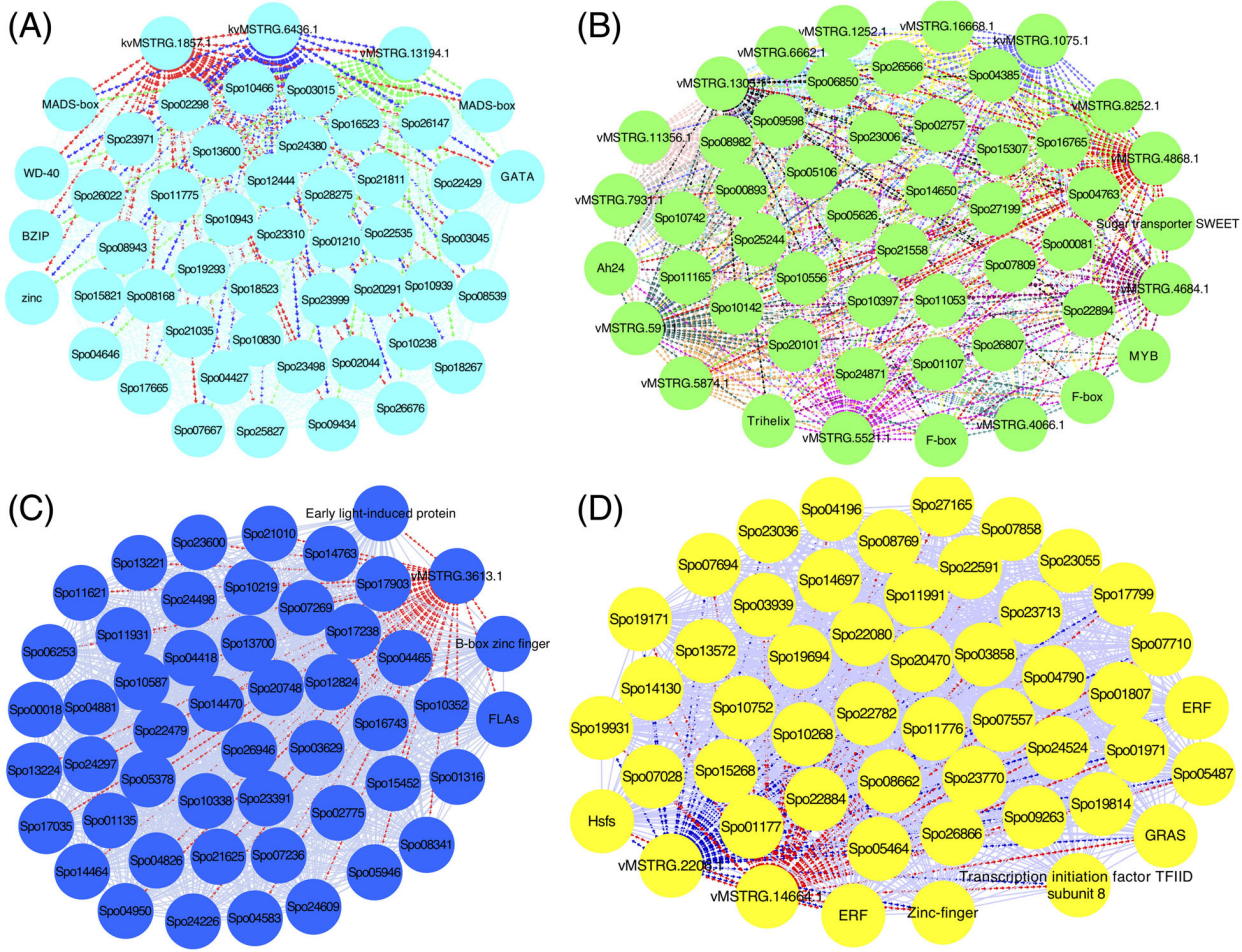
Co-expression network analysis of 50 hub genes from the three modules, including (**A**) turquoise, (**B**) green, (**C**) blue, and (**D**) yellow. Different colors represent different modules. Nodes represent the gene and edges indicate the interaction between genes

To identify the central and highly connected genes in the modules, we selected 50 top genes in the four main modules and visualized these genes by Cytoscape (Fig. 10). In the turquoise module, we identified 47 protein-coding genes and 3 lncRNAs. In this module, several well-known TFs, including MADS-box, GATA, and BZIP, were found in the list of the protein-coding genes. Further, we found UDP-glycosyltransferases 1 (*UGT1*, Spo22429) genes, which play a key role in regulating flowering time via the flowering repressor *FLC*. The stringent analysis of the expression pattern indicated UGT1 was highly down-regulated in accession Viroflay at the vegetative stage. In contrast, it showd up-regulation pattern in accession Kashan at the vegetative stage. Besides, GATA transcription factor, which delays flowering through repressing of SUPPRESSOR OF OVEREXPRESSION OF CONSTANS1 (*SOC1*) expression, was found in the turquoise module with a higher expression in the vegetative stage of accession Viroflay. Strikingly, we found lncRNAs in the turquoise module closely linked to *UGT1* and TFs, indicating that these lncRNAs may function as regulators of these genes in spinach.

In the green module, we identified 36 protein-coding genes and 14 lncRNAs. In this module, we found Sugar transporter SWEET gene, which is reported to be associated with flowering-time control [56]. Moreover, we identified SQUAMOSA PROMOTER BINDING-LIKE PROTEIN 6 (*SPL6*), a candidate gene related to the aging pathway. Among the hub genes of the green module, we reported two TFs, including Myb-like and Trihelix, and two F-box proteins. More investigation on blue and yellow modules identified one and two lncRNAs among the 50 top genes of these modules, respectively. UGT and Early light-induced protein were identified as putative genes related to the flowering in the blue module.Network analysis pinpointed UGT and several putative TFs, including three Ethylene-responsive (ERF2:Spo20470, ERF5:Spo25961, ERF510:Spo06220), Zinc-finger, GRAS family, and Heat shock in the yellow module that could be important regulators of bolting in spinach.

### qRT-PCR validation of selected DE-lncRNAs from each module

**Fig. 11:**
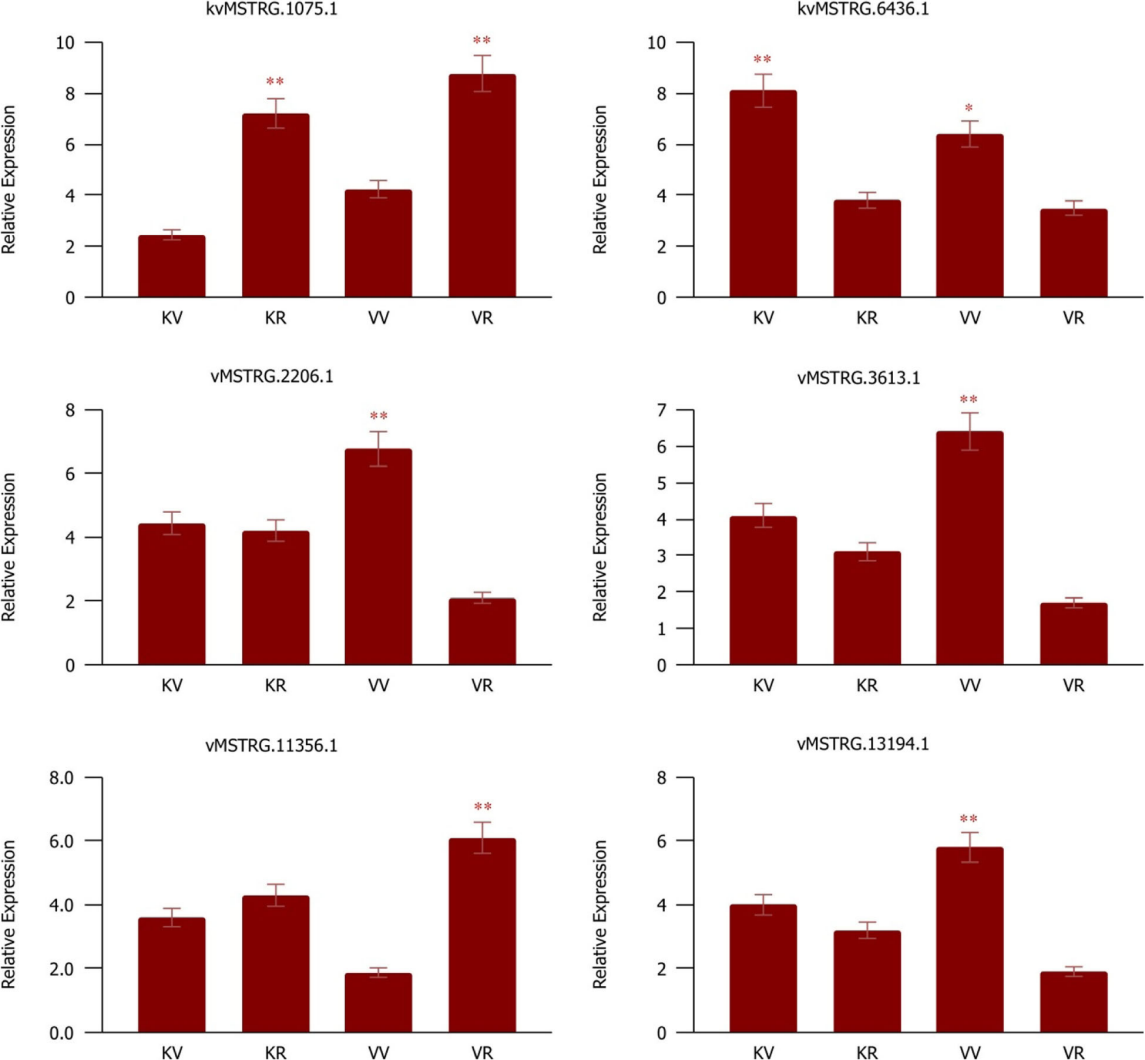
The relative expression of selected lncRNAs determined by qPCR in two accessions Kashan and Viroflay at two vegetative and reproductive development stages. KV, KR, VV, and VR represent Kashan-vegetative, Kashan-reproductive, Viroflay-vegetative, and Viroflay-reproductive, respectively. Here the data represented are relative quantification (RQ) values of gene expression. Bars represent the mean of three biological and technical replicates. ** or * indicate significant differences using Student *t-test* with *p*-value < 0.01 or < 0.05, respectively

To validate the expression of DE-lncRNAs obtained through RNA-Seq, we selected six hub DE-lncRNAs identified in the four main modules. These DE-lncRNAs were closely linked to the putative genes related to the flowering such as TFs, *FLC*, and *UGT1*, indicating that these lncRNAs may function as regulators of these genes in spinach. The qPCR results showed that the expression of the lncRNAs, including vMSTRG.13194.1, vMSTRG.2206.1, and vMSTRG.3613.1 were significantly up-regulated only in the vegetative stage of accession Viroflay compared to the reproductive stage (Fig. 11), whereas kvMSTRG.6436.1 was significantly up-regulated in the vegetative stage of both accessions. On the contrary, the expression of the lncRNA kvMSTRG.1075.1 was significantly down-regulated in the vegetative stage of both accessions compared to the reproductive stage. vMSTRG.11356.1 was found to be up-regulated only in the reproductive stage of accession Viroflay. Importantly, to evaluate concordance in gene expression between RNA-seq and qPCR, we focussed our analysis on genes expression correlation between normalized RT-qPCR Cq-values and log transformed RNA-seq expression values (R^2^ = 0.93), indicating that our analysis of the RNA-seq data was reliable. In this regards, the results of expression pattern of all six lncRNAs in qPCR were similar to the results of transcriptome analysis and didn’t find any dissimilarities between them.

## Discussion

Bolting or early-flowering is considered the main threat to the productivity of vegetable crops, especially spinach as a commercial and nutritional crop, and to date, limited studies have revealed a few molecular aspects of flowering mechanisms in this crop [13, 57]. Alongside protein-coding genes, lncRNAs play important roles in plant developmental pathways such as the transition from the vegetative to the flowering stage and flowering time control through regulating the expression of genes at the transcriptional or post-transcriptional level [21]. Unraveling the association between lncRNAs and functionally related genes requires potential investigations. One way to extract this information could be facilitated through sub-categorizing of protein-coding genes and lncRNAs in gene co-expression networks because it has been demonstrated that genes in the same module are presumably contributed in similar biological processes [58]. Hence, we identified the lncRNAs, analyzed the expression profile of two early and late-bolting spinach accessions, compared their expression levels in leaf tissues before and after flowering and confirmed them in flowering tissues using deep transcriptome data. We also predicted the potential function of lncRNAs and their cis-acting and trans targets genes. Additionally, we acquired the lncRNA-related pathway and gene ontology information and constructed a co-expression network based on the interaction between lncRNA and mRNA. In this study, a total of 1,141 potential lncRNAs were identified, 111 of which were significantly DE between vegetative and reproductive stages. While analyzing the characteristics of lncRNAs, we found that the number of lncRNA in our study was higher than lncRNAs reported by Li et al., 2020 [57]. As shown in Fig. 2, the expression profile of DE-lncRNAs was distinct between Kashan and Viroflay, confirming the accession-specific expression of lncRNA. Hence, lncRNAs with different expressions in the accession with different bolting times may be involved in the flowering time control. In further investigation, we examined the conservation and expression of identified lncRNAs in flower tissues. According to the results, ~ 68 % lncRNA identified in leaf tissues, were conserved and expressed in flower tissue. However, spinach lncRNAs were not well-conserved and expressed at the lower level compared with protein-coding genes, consistent with other studies examining plant lncRNAs [14, 59–61]. Notably, no homologous lncRNA was identified in spinach compared with Arabidopsis in the NONCODE database [62]. These results suggest that lncRNAs identified in our study were not conserved with currently known lncRNAs among different plant species, possibly due to (1) lncRNAs are not constrained by codon usage [61], (2) short motifs are not easily identifiable by BLAST search [59], and (3) lncRNAs may directly interact with RNA-binding proteins through conserved secondary structures [32]. This finding is similar to the results observed for lncRNAs in other plant species [14, 60, 61]. Among the main regulatory pathways for flowering control, including the photoperiod, vernalization, autonomous, age, and gibberellin pathways, vernalization is well-known to involve lncRNAs in the regulation of *FLC* expression. Recent studies have demonstrated that at least two types of lncRNAs, including COOLAIR (located at the 3’ end of the *FLC* locus) and COLDAIR, are involved in the modulation of *FLC* expression [26, 63–66]. Besides, genome-wide studies in *A. thaliana* have identified lncRNA CDF5 LONG NONCODING RNA (FLORE), which control flowering through regulating the expression *FLOWERING LOCUS T* (*FT*) [67]. However, no significant matches were obtained with COOLAIR. COLDAIR, and CDF5/FLORE among the all lncRNAs in this study. One possible explanation for this observation is that the regulatory mechanism of *FLC* and *FT* differ between spinach and Arabidopsis.

To improve our understanding of the functions and regulatory mechanisms of lncRNAs, we predicted cis-acting target genes of lncRNAs and DE-lncRNAs and performed the GO classification and KEGG enrichment analyses of the cis-acting target genes. The GO term classified in the biological process category was mainly derived from target genes located upstream of all lncRNAs and mostly associated with the accession Viroflay for DE-lncRNAs. Through GO functional enrichment analysis, we found that cis-acting target genes of DE-lncRNAs were enriched in terms including reproduction (GO:0000003), carbohydrate metabolic process (GO:0005975), and flower development (GO:0009908), which are directly related to the flowering regulation. It is proposed that carbohydrates provide energy sufficient for inflorescence development [10, 68]. Moreover, the cis-acting target genes of lncRNAs were significantly enriched in 15 pathways via KEGG analysis. The results of the KEGG enrichment analysis revealed pathways including “phenylpropanoid biosynthesis”, “plant hormone signal transduction”, “Starch and sucrose metabolism”, “MAPK signaling pathway”, and “circadian rhythm”, which are essential for the growth and flower development. These findings indicate that lncRNAs identified in this study are mainly associated with flowering-related pathways.

In addition to finding cit-targets genes, the novel interactions between DE-lncRNAs and flowering-related genes were successfully unraveled in both Kashan and Viroflay accessions using prediction of DE-lncRNAs’s trans target genes. For instance, an interplay between the bHLH transcription factor (*PIF4*) and several DE-lncRNAs especially those exhibited expression in both leaf and flower tissues was identified in early bolting Kashan accession. The prominent role of this gene has been displayed in ambient temperature-mediated flowering time [29]. It is proven that numerous higher plants increase their growth rate and accelerate the floral transition in response to warmer ambient temperatures. *PIF4* has been found to activate *FT* through the temperature dependent binding to *FT* promoter [69], which is known as a key gene involved in flower induction of many plant species [70]. In the present study, the upregulation of *PIF4* was observed in vegetative stage of Kashan compared to the reproductive stage. This result suggested the positive regulatory role of this gene along with its associated DE-lncRNAs including, MSTRG.1857.1 and MSTRG.8334.1 in a participatory manner on floral transition. In contrast, the other regulatory interaction was unveiled through mediating regulation by MSTRG.5258.1, MSTRG.7253.1, and MSTRG.1075.1 on *PIF4* expression. On the basis of these findings, we can unveil flowering acceleration in Kashan through the PIF4/lncRNAs interaction.

In late bolting Viroflay accession, the novel relationship was revealed between *SNAT2* and DE-lncRNAs which were commonly shared between leaf and flower tissues. Generally, *SNAT2* contributes to melatonin synthesis, and by this function modulates various physiological responses. The key role of this gene has been detected in the GA-related flowering pathway and suppression of *FT* gene expression as well [71]. Lee et al. (2019) indicated that knockout of *AtSNAT2* led to reduction of melatonin levels and also flowering delay. They attributed delay of flowering time to decrement in the expression levels of gibberellin biosynthetic genes like ent-kaurene synthase (*KS*) and reduction in *FT* expression levels. According to this study, the upregulation of *SNAT2* in the Kashan vegetative stage presumably revealed its positive regulatory role on early floral transition of this accession in comparison with the Viroflay, which exhibited the downregulation of *SNAT2*. Additionally, our results unraveled *SNAT2/*DE-lncRNAs (MSTRG.6436.1, MSTRG.3613.1, and MSTRG.13194.1) interaction-mediated flowering time regulation in Viroflay. Our results in Viroflay pointed out the interaction between another flowering-associated gene, *HUA1*, and common DE-lncRNAs in both tissues of leaf and flower. *HUA1*, as an RNA-binding protein has been turned out to contribute in regulation of MADS-box floral homeotic gene AGAMOUS (*AG*) along with other *AG* mRNA processing factors including *FLOWERING LOCUS WITH KH DOMAINS* (*FLK*), *PEPPER* (*PEP*) and *HUA ENHANCER 4* (*HEN4*) genes. It has been illustrated that the partnership possibly led to floral organ identity and floral meristem determinacy [72, 73]. In our study, the probable collaborative-based interaction was observed between *HUA1* and its relevant DE-lncRNAs including MSTRG.19145.1, MSTRG.13194.1, MSTRG.9452.1 and MSTRG.7434.1, which emerged as over-expressed genes in vegetative stage of both accessions. This result might disclose their positive regulatory role in stimulating spinach flower induction and development. Additionally, *ELF6* was predicted as another downstream-trans target of DE-lncRNAs in Viroflay. This gene is identified as a leading gene negatively involved in the regulation of flowering time through repressing the photoperiodic floral regulatory pathway [74]. Jeong et al. (2009) displayed this gene directly repressing *FT* gene and inhibiting precocious flowering in Arabidopsis [75]. The downregulation of *ELF6* and the opposite expression pattern of some associated controlling lncRNAs including MSTRG.1857.1, MSTRG.1953.1, MSTRG.8334.1, and MSTRG.14944.1 in vegetative stage of both Kashan and Viroflay accessions can unveil the regulatory mechanisms-mediated lncRNAs mitigating inhibitory effects of *ELF6* on *FT* expression.

Another novel regulatory interaction was found between *PKL* and pertinent De-lncRNAs including, MSTRG.1857.1, MSTRG.1953.1, and MSTRG.14944.1 in Viroflay. *PKL* is well-known as a CHD3 chromatin-remodeling factor that promotes flowering initiation (Henderson et al., 2004). Fu et al. (2016) revealed the regulatory interaction between *PKL* and *LFY*gene which regulates its own downstream floral meristem genes. The negative regulatory role of *ORTH2* on flowering time has been proved in Arabidopsis [76]. Kraft et al. (2008) indicated that transgenic plants over-expressing *ORTH2* exhibited a late-flowering phenotype [77]. For this gene the opposite changes of expression pattern was detected between two Kashan and Viroflay accessions in vegatative stage compared with the reproductive stage. This difference in expression pattern can divulge the presumptive role of *ORTH2* on flowering time as the distinct phenotypic attribute between two accessions. Interestingly, in Viroflay the probable lncRNA-mediated modulation of *ORTH2* expression was discovered through expression analysis of DE-lncRNAs such as MSTRG.575.1, MSTRG.5258.1 and MSTRG.1075.1 that were predicted as regulators of upstream flowering-related trans target genes. *CK2* as a protein kinase contributes to the modulation of the circadian clock and photoperiod pathway. It has been demonstrated that *CK2* positively controls flowering time by repressing the expression of *FLC* [78]. In our study the positive regulatory role of *CK2* was also revealed in Viroflay. Moreover, the potential regulatory role of MSTRG.575.1, MSTRG.1252.1, MSTRG.1305.1 was observed on overexpression of *CK2*, resulting in flowering time regulation. Based on our expression data, *PHL*, a nuclear proteins with an important role in regulating photoperiodic flowering and accelerates flowering through physical interactions with phytochrome B (*phyB*) and CONSTANS (*CO*) [79], emerged as down-expressed genes in vegetative stage compared to the reproductive stage. Since this observed expression pattern in the vegetative stage might prolong flowering initiation, we can conclude the probable pivotal role of this gene in induction of late bolting phenotype of Viroflay. Besides, our results detected PHL/MSTRG.1856.1 interplay-mediated flowering time regulation in Viroflay. Similar to *PHL*, *GASA5* emerged as the overexpressed gene in the vegetative stage. Since the genetic evidence has explained that *GASA5* induces late flowering by suppressing the expression of the *FT* and *LFY* as major flowering-time genes [80, 81], this result suggests the possible role of this gene in Viroflay’s late bolting phenotype excitation as well. Additionally, the collaborative-based link was discovered between *GASA5* and its associated DE-lncRNAs including MSTRG.1857.1, MSTRG.1953.1, MSTRG.6436.1, and MSTRG.706.1.

Numerous studies have reported that plant lncRNAs could act as eTMs by binding to specific miRNA, competing with the target mRNA of miRNA and thus blocking the cleavage and alleviating the repression of its target gene [82–84]. In our study, two DE-lncRNAs were identified as eTMs for two miR172 and miR167, which play critical roles in flowering time [85, 86]. Previous researches have demonstrated that miR172 could affect the expression level of APETALA-2 (*AP2*) [85] and also target some TFs [86, 87]. Here we found three genes encoding AP2/ERF as targets of miR172, which known to have a potential impact to positively/negatively regulate various processes such as control of metabolism, growth, and development, as well as flowering regulation through the photoperiod pathway [88]. These results indicate that the lncRNA-miRNA (MSTRG.16566.1-mir172) pairs might be important novel regulatory components in the flowering/bolting of spinach.

Once we identified final DE-lncRNAs and DEGs sets, WGCNA was then carried out to construct related-flowering regulatory networks in spinach with the aim to elucidate the interaction of protein-coding genes and lncRNAs and identify key hub genes. Among identified co-expression modules, the turquoise module contained the largest number of DE-lncRNAs, including 3 unique DE-lncRNAs in Kashan, 23 unique DE-lncRNAs in Viroflay, and 6 common DE-lncRNAs between both accessions. In this module, we identified *Flowering locus T-like 1* (*FLT1*), *EARLY FLOWERING 4* (*ELF4*), *CONSTANS-LIKE 1* (*COL1*), and *AGAMOUS-LIKE* (*AGL*) which are well-known genes in controlling circadian rhythms and flowering time [89, 90]. Besides, we found other crucial flowering-related genes including UDP-glycosyltransferase enzymes (*UGTs*): regulates flowering time via the flowering repressor *FLC* [91], Serine/arginine-rich (*SR*): delays flowering time [8], Sugar transporter: acts downstream of *FLT* during the floral transition [56], Transducin/WD-40: an important gene in regulating flowering time [92], Dof protein: plays a key role in photoperiodic flowering time [93], Glutaredoxin: affects flowering time [94], Frigida-like: can modulate flowering time [15], Pentatricopeptide repeat protein: affects flowering time [95], Glutathione S-transferase: plays roles in regulating flowering time in response to light [96], F-box protein: promotes flowering [97], and Ubiquitin-conjugating enzyme which is involved in the regulation of flowering time [98]. In the turquoise model, TFs such as ERF, BHLH, AP2/B3-like, BZIP, MYB, WRKY, GATA, NAC domain, Zinc finger, MADS-box, and CCAAT-binding factor were also identified as the paramount regulators of flowering [10, 11, 99–102]. Previous studies have shown that most of these TFs could act as the target of lncRNAs [33, 61].

By searching against the genes list of the blue module, we found that 5.1 % of the member transcripts were DE-lncRNAs, containing two unique DE-lncRNAs in Kashan, 16 unique DE-lncRNAs in Viroflay, and 2 common DE-lncRNAs between both accessions. The core flowering genes in this module were *FLC* and *FLOWERING PROMOTING FACTOR 1* (*FPF1*), along with flowering-related genes including VQ motif-containing protein [103], E3 ubiquitin-protein ligase [104], Ubiquitin-conjugating enzyme h [105], UDP-glycosyltransferase [91], Transducin/WD40 [92], CASP-like [106], Pentatricopeptide repeat [95], F-box protein [97], B-box zinc finger [105], and Sugar transporter [56], which play important roles in regulating flowering time. TFs in the blue module included GRAS, GATA zinc finger, MYB, ERF, and NAC domain. In the black module, five DE-lncRNAs were recognized as the flowering-associated lncRNA, together with four well-known TFs including zinc finger, F-box, ERF, WRKYand other neighboring co-expressed mRNAs such as sugar transporter and UDP-glycosyltransferase. Interestingly, most of the genes mentioned above, importantly *ELF3* and *COL1*, were also identified in the other modules.

Furthermore, the KEGG analysis of genes from two turquoise and blue modules supports that the candidate target genes of lncRNAs existing in these modules may be involved in the regulation of circadian rhythm. Altogether, we found that novel identified lncRNAs in this study may be involved in the regulation of key flowering-related genes and related pathways like circadian rhythm.

## Materials and methods

### Description of transcriptome datasets

Two transcriptome datasets [13] generated from leaf tissues of two different accessions at vegetative and flowering stages were used to identify bolting-related lncRNAs. Our previous study has described sampling, RNA extraction, and sequencing methods [13]. In brief, seeds of two accessions Viroflay and Kashan as late and early flowering spinach samples, respectively, were sown in plastic pots with sterilized soil and grown in a growth chamber under spring growth conditions for 3 months at Isfahan University of Technology, Isfahan, Iran, in March 2018. Total RNAs were extracted from leaf samples using the DENAzist column RNA isolation kit. After quantification, RNAs were sent to Personalbio (Shanghai, China) company for cDNA library preparation and sequencing. The sequencing was done on an Illumina platform with 150 bp paired-end readers. In the current study, one dataset included six samples of accession Kashan representing three biological replicates at each developmental stage. The second dataset included the same number of samples at the same stages representing the accession Viroflay. Datasets were deposited at Sequence Read Archive (SRA) at NCBI with accession numbers PRJNA630139.

### Identification of unannotated transcripts

The paired-end RNA-Seq reads were subjected to quality checks and filter out low-quality reads and adaptor sequences using FastQC (https://www.bioinformatics.babraham.ac.uk/projects/fastqc/) and Trimmomatic v0.30 program [107](Bolger et al., 2014), respectively. Trimmomatic software was set to keep reads longer than 50 bp with a minimum Phred score of 30. After trimming, the clean reads from each library were mapped with the spinach reference genome version 1 located in SpinachBase[48, 108] using STAR v2.7.1 [109]. To derive unannotated transcripts, the StringTie v2.0.6 [110] was used to assemble all transcripts, then StringTie’s merge was applied to combine the assembled transcripts and create a unique set of transcripts. In this step, the output file of StringTie’s merge was compared with the gene annotation file (GTF file) of the reference genome using gffcompare to classify transcripts in different classes. Then, unannotated transcripts were extracted from gffcompare output based on the class codes, including “u” (intergenic lncRNAs), “x” (anti-sense lncRNAs), “i” (intronic lncRNAs), “o” (generic exonic overlap lncRNAs with reference transcripts), and “e” (single exon transfrag overlapping a reference exon). Finally, BEDTools v2.29.2 [111] was used to extract the sequences from the spinach reference genome by defining unannotated transcripts names in a BED file.

### LncRNA prediction pipeline

To identify potential lncRNAs, the unannotated transcripts were subjected to some filtering approaches (Fig. 1 A). First, the unannotated transcripts with CPM (counts per millione) < 1 and less than 200 nucleotides were excluded. Second, FEELnc v.0.2 [112] with a shuffle mode (-m “shuffle”) was used to infer potential lncRNAs from unannotated transcripts. Third, the coding potential of predicted lncRNAs was further evaluated using coding potential calculator (CPC2) software [113]. Fourth, tRNAscan-SE 2.0 [114] and Barrnap 0.9 (https://github.com/tseemann/barrnap) were applied to filter out possible transfer RNAs (tRNAs) and ribosomal RNAs (rRNAs). Fifth, remaining transcript sequences were then inputted into CREMA (available at www.github.com/gbgolding/crema) [47] to increase specificity and accuracy of lncRNA prediction and ranking. Since the definition of lncRNA is rather arbitrary, various tools have been developed to evaluate the coding potential of transcripts and distinguish non-coding RNAs from protein-coding ones using machine learning approaches. Hence, we applied additional tools namely RNAplonc [115] to check and evaluate identified lncRNAs. A search with BLAST tools was performed against the UniProt release 2020-02, Pfam release 28.0, and Rfam 14.4 database to remove transcripts encoding any conserved protein and domain. Due to the presence of known lncRNAs in some existing plants, we compared the identified lncRNAs in this study with the lncRNAs available in GreeNC [44], CANTATAdb v2.0 [45], lncRNAdb v2.0 [116], and PLncDB v2.0 [43] databases by alignment using BLASTn to assess the conservation of spinach lncRNAs and make this set more credible. BLASTN search was performed at the criteria of e-value 1e-5, identity > 70 %, and query coverage > 30 %.

### Mining of differentially expressed genes (DEGs)

For differential expression analysis, gene read-count data matrices were produced from assembled transcripts with python script prepDE.py. Then, the IDEAMEX website [117] was used to call all DEGs through DESeq2 [118] software with ‘‘FDR ≤ 0.05, logFC > = 2 and CPM = 1” parameters.

### Tracking of identified lncRNAs in the spinach flower tissues

To investigate whether lncRNAs identified in the leaf samples are also conserved and expressed in flower tissues, we employed the transcriptome dataset of young leaf (YL) and female flower samples of *s. oleracea* in five different developmental stages including, the formation of ovary (FO), sepal primordia and carpel primordium (SPCP), floral meristem (FM), ovule differentiates within the ovary (ODVO), and ovule matures (OM), available in the European Nucleotide Archive (ENA, https://www.ebi.ac.uk/ena) under the bioproject accession number PRJNA649901. To identify lncRNAs expressed in flower transcriptome, all data processing steps including, quality control, trimming, mapping, identification of unannotated transcripts, and subjecting to lncRNA filtering pipeline were performed as described above in “Identification of unannotated transcripts” and “LncRNA prediction pipeline” sections. Subsequently, potential lncRNAs identified in leaf tissues of Viroflay and Kashan accessions at vegetative and reproductive stages were compared to those identified lncRNA in flower samples using BLASTn search with the criteria of e-value 1e-20 and minimum alignment length of 200 bp. Moreover, the differential expression analysis of lncRNAs identified in young leaf and aforesaid flower developmental stages was carried out as mentioned before in “Mining of differentially expressed genes (DEGs)” section.

### Prediction of DE-lncRNA’s trans target genes using LncTar

The interaction between DE-lncRNA and trans-regulated target genes as DEGs was investigated by using LncTar [119]. For this purpose, DEGs located in 100 kb upstream and downstream of potential DE-lncRNAs (cis target genes) identified in Kashan and Viroflay accessions were imported as inputs to LncTar, and tools was implemented with default parameters. Accordingly, in this study the biological function of genes located 100 Kbp upstream and downstream of lncRNAs as cis-regulated potential target genes were investigated via Gene Ontology (GO) and Kyoto Encyclopedia of Genes and Genomes (KEGG; http://www.genome.jp/kegg/) pathway enrichment analysis [120].

### Identification of DE-lncRNAs that act as eTMs

To identify DE-lncRNAs that can act as eTMs of miRNAs, all DE-lncRNAs candidates were used to predict miRNA mimic sites using the psMimic software [121]. In this way, all previously-known plant miRNAs from the miRBase database (http://www.mirbase.org/; release 22.1, October 2018) [122] were downloaded and clustered using CD-HIT-EST [123] with the following parameters: c = 1, n = 10, d = 0, and M = 16,000. Putative target genes of the predicted miRNAs that had mimicry with lncRNAs were identified using the plant miRNA target prediction online software psRobot with moderate parameters [124].

### Detection of lncRNAs expression by qRT-PCR

To verify the abundance of the predicted lncRNAs, the expression levels of six lncRNAs were detected through real-time quantitative PCR (qRT-PCR). Hence, total RNAs were extracted using the DENAzist column RNA isolation kit and reverse-transcribed into cDNA using RevertAid First Strand cDNA Synthesis Kit (Thermo Fisher, Co., USA), according to the manufacturer’s instructions. Then, specific primers (Table 3) and SYBR Green PCR Master Mix (BioFACT, Korea) were used to perform qRT-PCR in three technical replicates using an ABI system (ABI ViiA 7 Real-time PCR) in a 20 µL final volume. Actin and GAPDH housekeeping genes were used as internal reference genes, and the data were analyzed by the 2-ΔΔCt method.

**Table 3:** LncRNAs and primers set used for qRT-PCR analysis

Gene IDs	Primers Sequence	Product Size
vMSTRG.3613.1_F	CCTTGGTGGAGGCTTATTGA	197
vMSTRG.3613.1_R	TTCCTCCTCCAGTTCACCAC	
vMSTRG.13194.1_F	TCACCCTCTGACCAAAAAGC	209
vMSTRG.13194.1_R	TTTGAGGCCTTAGGCAAAGA	
kvMSTRG.6436.1_F	CATTTTTGCGCACTTGCTAA	206
kvMSTRG.6436.1_R	GGTGGAGGAAGATGGTGAGA	
vMSTRG.11356.1_F	GCTTTTGGTTTCGCTCAAAG	227
vMSTRG.11356.1_R	AGAGCAGTAGGTGGCAAGGA	
kvMSTRG.1075.1_F	GCATAACCGCACATCAACAC	165
kvMSTRG.1075.1_R	TAGTGTAACCGGCCAAGACC	
vMSTRG.2206.1_F	GGAAAAATTGGAACGAAGCA	164
vMSTRG.2206.1_R	GCCCGAAAATAAGTCAGCAG	

### Coding/non-coding gene co-expression network

To detect similar expression patterns between the lncRNAs and mRNAs, we used the WGCNA [125] R software package. First, the normalized fragments per kilobase of transcript per million fragments mapped (FPKM) values were used as the input file for significantly differentially expressed lncRNAs and mRNAs. Based on log2 (FPKM + 1) values, a similarity matrix was generated by calculating Pearson’s correlation between all pairs of genes and then transformed into an adjacency matrix. According to the scale-free topology criterion [126], soft power was set to 9. After that, the topological overlap measure (TOM) and corresponding dissimilarity (1-TOM) were calculated using the adjacency matrix. Then, the modules, which are clusters of highly interconnected genes, were identified by hierarchical clustering of 1-TOM and the DynamicTree Cut algorithm [125]. Additionally, correlations among gene expression modules and phenotypic traits were investigated; day to flowering [46] was chosen as our interesting trait. Modules that were significantly correlated with the trait (r > 0.7, *P-value* < 0.05) were identified; and genes in significant modules were then exported for further analysis. Finally, to determine the genes that are highly connected in the modules, we selected the 50 top hub genes through the Cyto-Hubba plugin [127] and visualized these genes by Cytoscape [128] as an unsigned network.

## Supplementary Information

**Additional file 1:**

## Data Availability

The transcriptome sequencing data have been obtained from the NCBI Sequence Read Archive (https://www.ncbi.nlm.nih.gov/bioproject/PRJNA630139) under accession number PRJNA630139.
