# Supplementary material for: Global identification of long non-coding RNAs involved in the induction of spinach flowering
Source: BMC Genomics. 2021 Sep 30;22:704. doi: 10.1186/s12864-021-07989-1 (PMC8482690; doi:10.1186/s12864-021-07989-1)
Supplement: Supplementary file 1 — Additional file 1: [file 12864_2021_7989_MOESM1_ESM.docx]

- **Global identification of long non-coding RNAs involved in the induction of spinach flowering**

Fatemeh ghorbani^1^, Reza Abolghasemi^2^, Maryam Haghighi^2^, Nematollah Etemadi^2^, Shui Wang^3^, Aboozar Soorni^1^*

^1^Department of Biotechnology, College of Agriculture, Isfahan University of Technology, Isfahan, Iran

^2^Department of Horticulture, College of Agriculture, Isfahan University of Technology, Isfahan, Iran

^3^College of Life and Environmental Sciences, Shanghai Normal University, Shanghai, China

*Correspondence: [soorni@iut.ac.ir](mailto:soorni@iut.ac.ir)

**
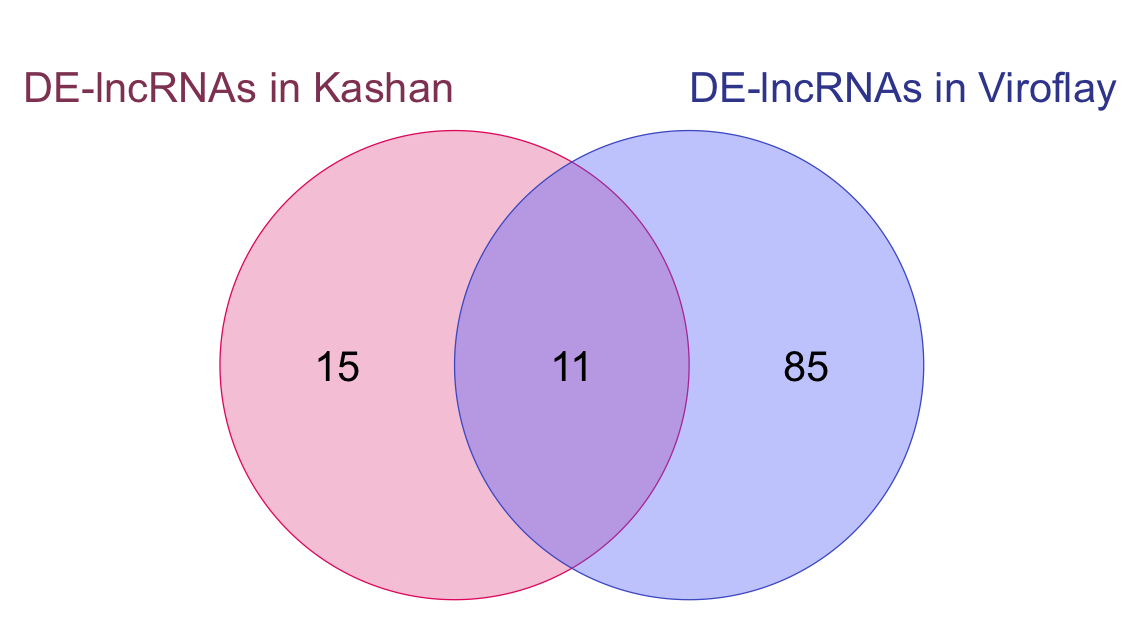
**

**Figure S1** Venn diagram showing the distribution of unique and common DE-lncRNAs in accessions under stages comparison.

**Table S1** The predicted lncRNAs using computational screening against databases.

| Spinach lncRNA ID | Database | lncRNA ID | Homologous species |
| --- | --- | --- | --- |
| chr2:46856781-46859239 | CANTATAdb | CNT20121134 | *Trifolium pratense* |
| chr4:61653984-61660101 | CANTATAdb | CNT2046539 | *Chenopodium quinoa* |
| chr5:56447013-56452482 | CANTATAdb | CNT2046628 | *Chenopodium quinoa* |
| chr1:29295909-29297294 | CANTATAdb | CNT2048034 | *Chenopodium quinoa* |
| Super_scaffold_229:276047-280716 | CANTATAdb | CNT2048127 | *Chenopodium quinoa* |
| Super_scaffold_229:276047-280716 | PLncDB | SLYC_LNC000377.2 | *Solanum lycopersicum* |
| chr1:41329767-41330372 | CANTATAdb | CNT2048767 | *Chenopodium quinoa* |
| SpoScf_28366:231-1211 | CANTATAdb | CNT2050621 | *Chenopodium quinoa* |
| chr6:23539897-23540797 | CANTATAdb | CNT2050939 | *Chenopodium quinoa* |
| SpoScf_00137:670930-673704 | CANTATAdb | CNT2051452 | *Chenopodium quinoa* |
| SpoScf_22627:1-1498 | CANTATAdb | CNT2051452 | *Chenopodium quinoa* |
| Super_scaffold_45:2175640-2177931 | CANTATAdb | CNT2051452 | *Chenopodium quinoa* |
| SpoScf_46130:1-863 | CANTATAdb | CNT2052620 | *Chenopodium quinoa* |
| chr4:12306220-12306932 | CANTATAdb | CNT2053362 | *Chenopodium quinoa* |
| SpoScf_01003:55322-58020 | CANTATAdb | CNT2054530 | *Chenopodium quinoa* |
| SpoScf_01037:51886-53051 | CANTATAdb | CNT2056968 | *Chenopodium quinoa* |
| SpoScf_02079:60070-63679 | CANTATAdb | CNT2060981 | *Chenopodium quinoa* |
| chr2:42077964-42083402 | CANTATAdb | CNT2061120 | *Chenopodium quinoa* |

**Table S2** The DE-lncRNAs that were predicted as eTMs of in spinach

| miRNAs ID | LncRNAs ID | Accession name |
| --- | --- | --- |
| ath-miR-1888a | chr4:88808719-88810196 | Kashan |
| bma-miR-5846 | chr5:14399999-14401098 | Viroflay |
| bmo-miR-2818 | SpoScf_00978:37871-39328 | Viroflay |
| bta-miR-2321 | chr4:71442586-71444817 | Viroflay |
| bta-miR-2371 | SpoScf_02234:45257-48361 | Viroflay |
| bta-miR-6528 | SpoScf_02008:95154-96342 | Kashan |
| cbn-miR-58b | SpoScf_01768:73161-75908 | Viroflay |
| cbr-miR-2224 | SpoScf_00655:119957-122825 | Viroflay |
| cbr-miR-54a-5p | chr4:74504761-74510062 | Viroflay |
| cgr-miR-1903 | chr1:27674540-27675225 | Viroflay |
| cin-miR-4122-3p | SpoScf_01143:100750-101777 | Viroflay |
| cin-miR-4188-5p | SpoScf_01473:60000-60687 | Viroflay |
| cin-miR-4192-3p | SpoScf_01002:80279-82240 | Viroflay |
| cja-miR-9981 | Super_scaffold_58:2221813-2225483 | Viroflay |
| cre-miR-1168.2 | SpoScf_01668:122743-123451 | Kashan |
| dme-miR-2491-3p | chr3:97868764-97870199 | Viroflay |
| dme-miR-2491-3p | SpoScf_01589:134256-139853 | Viroflay |
| dme-miR-4971-5p | SpoScf_00409:167348-168202 | Viroflay |
| dre-miR-737-5p | SpoScf_00924:202781-204736 | Viroflay |
| egr-miR-10266-3p | chr1:7754734-7756818 | Kashan |
| esi-miR-8628 | Super_scaffold_60:75828-76959 | Both |
| gga-miR-1641 | SpoScf_00301:93864-99818 | Viroflay |
| gga-miR-1814 | SpoScf_00887:76457-77665 | both |
| gga-miR-1816 | chr1:42746296-42747861 | Viroflay |
| ggo-miR-198 | SpoScf_07684:1091-3080 | Kashan |
| gma-miR-1522 | chr3:103701194-103702200 | Viroflay |
| gmo-miR-219-3p | chr6:14925090-14925611 | Viroflay |
| hsa-miR-12114 | SpoScf_26871:1-657 | Viroflay |
| hsa-miR-1279 | chr4:21682463-21686701 | Viroflay |
| hsa-miR-1297 | Super_scaffold_229:276047-280716 | Viroflay |
| hsa-miR-2052 | SpoScf_02662:24638-29802 | Viroflay |
| hsa-miR-3123 | SpoScf_02005:6917-9875 | Viroflay |
| hsa-miR-320a-5p | SpoScf_02135:4093-4910 | Viroflay |
| hsa-miR-4261 | SpoScf_03215:5609-9909 | Viroflay |
| hsa-miR-4279 | chr3:16376177-16378098 | Viroflay |
| hsa-miR-4291 | SpoScf_01824:80635-81801 | Viroflay |
| hsa-miR-4297 | Super_scaffold_27:27310-31474 | Viroflay |
| hsa-miR-4317 | SpoScf_03024:5971-10132 | Viroflay |
| hsa-miR-4455 | SpoScf_51672:116-782 | Both |
| hsa-miR-4487 | chr5:33841798-33843970 | Viroflay |
| lja-miR-7536a | chr4:80678657-80682568 | Viroflay |
| mdo-miR-12362-5p | Super_scaffold_70:1572782-1573700 | Viroflay |
| mtr-miR-2676a | SpoScf_00630:36716-39390 | Viroflay |
| nve-miR-2033-5p | SpoScf_03835:617-4426 | Viroflay |
| oan-miR-1328 | SpoScf_00562:236276-239466 | Both |
| oan-miR-184 | Super_scaffold_181:1375003-1379159 | Viroflay |
| ola-miR-29c | SpoScf_00842:2481-5885 | Viroflay |
| osa-miR-444a-3p.1 | SpoScf_02537:58681-61670 | Viroflay |
| osa-miR-5537 | SpoScf_02115:52691-56489 | Viroflay |
| ppt-miR-1044-3p | SpoScf_03142:9164-11518 | Kashan |
| ppt-miR-1215 | chr3:111505538-111508869 | Viroflay |
| ptc-miR-167f-3p | chr4:38264207-38266517 | Viroflay |
| ptr-miR-891a | chr4:94807851-94808534 | Viroflay |
| pxy-miR-8500 | SpoScf_00041:105068-105971 | Viroflay |
| rgl-miR-5138 | SpoScf_00886:202935-204071 | Viroflay |
| rno-miR-193b-5p | chr4:7578897-7580540 | Viroflay |
| sfr-miR-10493-3p | chr6:31685765-31686675 | Viroflay |
| sja-miR-3486-5p | SpoScf_07371:737-2461 | Viroflay |
| sme-miR-2168-5p | SpoScf_03141:13735-17813 | Viroflay |
| sme-miR-2178 | SpoScf_02680:40931-44554 | Viroflay |
| sme-miR-71b-3p | SpoScf_01981:3950-6418 | Viroflay |
| ssa-miR-15b-5p | SpoScf_01678:128645-130733 | Viroflay |
| tca-miR-3842-5p | chr6:43117951-43119787 | Viroflay |
| tca-miR-3861-5p | SpoScf_02476:11671-13488 | Both |
| xla-miR-181b-2-3p | SpoScf_02594:55532-56847 | Viroflay |
| zma-miR-172a | chr4:76795449-76796456 | Kashan |
